# Epidemiology of Chronic Pain in Denmark and Sweden

**DOI:** 10.1155/2012/371248

**Published:** 2012-05-23

**Authors:** Julie Harker, Kim J. Reid, Geertruida E. Bekkering, Eliane Kellen, Malgorzata M. Bala, Rob Riemsma, Gill Worthy, Kate Misso, Jos Kleijnen

**Affiliations:** ^1^Kleijnen Systematic Reviews Ltd., 6 Escrick Business Park, Escrick, York YO19 6FD, UK; ^2^KJ Research, Rosemere, QC, Canada J7A 4N8; ^3^BeSyRe Bekkering Systematic Reviews, 2440 Geel, Belgium; ^4^Center for Evidence Based Medicine, Katholieke Universiteit Leuven, 3000 Leuven, Belgium; ^5^Leuven Centre for Cancer Prevention, University Hospital Leuven, 3000 Leuven, Belgium; ^6^2nd Department of Internal Medicine, Jagiellonian University Medical College, 31-066 Krakow, 8 Skawinska Street, Poland; ^7^School for Public Health and Primary Care (CAPHRI), University of Maastricht, 6200 MD Maastricht, The Netherlands

## Abstract

*Introduction*. Estimates on the epidemiology of chronic pain vary widely throughout Europe. It is unclear whether this variation reflects true differences between populations or methodological factors. Information on the epidemiology of chronic pain can support decision makers in allocating adequate health care resources. *Methods*. In order to obtain epidemiological data on chronic pain in Denmark and Sweden, we conducted a literature review of epidemiological data primarily on chronic noncancer pain, prioritising studies of highest quality, recency, and validity by conducting a systematic search for relevant studies. Following quality assessment, data were summarised and assigned to the research questions. *Results*. The prevalence of moderate to severe noncancer pain was estimated at 16% in Denmark and 18% in Sweden. Chronic pain impacts negatively on perceived health status, quality of life and is associated with increased cost. Despite using pain medications, a large proportion of chronic pain sufferers have inadequate pain control. There was a lack of high-quality and low-bias studies with clear inclusion criteria. *Conclusions*. In both Denmark and Sweden, chronic pain is a common health problem which is potentially undertreated and warrants attention of health care workers, policy makers and researchers. Future research should utilise clear reporting guidelines to assist decision and policy makers, in this important area.

## 1. Introduction

There are currently many differences in estimates of the prevalence of chronic pain in Europe, which typically range between 10–30% of the adult population, but studies have reported prevalence as high as 50% or as low as 2% [1–3]. It is unclear why this is the case but may be due to differences between patient populations or differences in definitions of chronic pain and assessment methodology used in epidemiological studies. Chronic pain is often reported to be more common in older age groups, lower income groups, and among women [4–7].

Although chronic pain represents a public health problem with an evident social and economical impact, there is a paucity of reliable data about its epidemiology, hindering an accurate estimation of the burden of this condition. Statistics rarely focus on chronic pain as a discrete entity. This ignores the insight of clinicians specialised in pain treatment: that chronic pain is considered a discrete entity in itself, with clear characteristics of symptoms, disability, and mental health aspects which are largely independent of the underlying disease.

Decision and policy makers in European countries make judgements on health budgets and patient segmenting and compared with cardiovascular disease, oncology, diabetes, and mental health; there appears to be limited appreciation about the importance of chronic pain. In a recent European White Paper by OpenMinds (the Opioids and Pain European Network of Minds) [8] the relatively high prevalence of chronic pain across Europe was highlighted, as well as the impact of chronic pain on everyday life to patients and health care providers and the reported undertreatment of chronic pain. Data about the epidemiology of chronic pain from prevalence to cost impacts would be useful for proper information and accurate decision making.

As part of a wider pan-European project [9–11], we obtained epidemiological data on research questions related to specific outcomes on chronic noncancer pain, based on a literature review of the most relevant scientific literature since 1995, carried out with the principles of systematic reviews in searching and critically summarizing the findings. We prioritized the appraisal of studies according to pertinence, recency, and validity, rather than summarizing the results of all studies that were identified. This paper summarises the reviews for Denmark and Sweden; which were initially conducted as separate literature reviews but are presented here combined due to the similarity of health care systems in Scandinavian countries [12] and the similarity of results.

## 2. Methods

This study was developed within the framework of a broad literature review about the epidemiology of chronic noncancer pain, across European countries including Denmark and Sweden, reviewing the scientific literature on the most recent epidemiological data on chronic pain. As far as practicable, we followed standardised processes and methods recommended by the Centre for Reviews and Dissemination (CRD) for systematic reviews [13], which were adapted slightly to allow us to select only the most relevant and up-to-date literature available. This was a narrative literature review and not a full Systematic Review of the available evidence.

Our extensive search strategy was developed to search for studies across European countries as part of a wider European project on the epidemiology of chronic pain, but for this paper only studies from Denmark and Sweden were utilised. In September 2009, the following databases were searched from 1995 to the end of August 2009: MEDLINE, EMBASE, The Cochrane Library, CRD Databases (DARE, HTA, and NHS EED), and GIN database. The strategy was limited to specific European countries and corresponding languages (German, French, Swedish, Spanish, Italian, Dutch, English, and Danish). Additionally we checked the reference lists of the studies deemed as relevant for the purposes of the review. Demographic data were taken from the Statistics Denmark website [14] and for Sweden from the European Commission Eurostat website [15]. The Medline search strategy is presented in Supplementary Materials, Appendix 1 available online at doi:10.1155/2012/371248. Full search strategies for all the resources searched are available by contacting the authors.

Our initial inclusion criteria were to include primary studies or systematic reviews of studies providing epidemiological data about noncancer chronic pain in Denmark and Sweden. Chronic pain was defined as painful conditions lasting at least 3 months or chronic diseases associated with pain, including musculoskeletal pain, neuropathic pain, fibromyalgia, osteoarthritis, and rheumatoid arthritis. We excluded studies with children and adolescents, conditions such as migraine and headache, angina pectoris, specific cancer pain, or pain associated with specific conditions (e.g., multiple sclerosis, Parkinson's disease, and gastroenterological conditions). However, due to a dearth of data in identified studies (e.g., some with cancer pain patients included and some with a pain population with unknown or mixed medical diagnoses), we modified these criteria to allow for studies with mixed pain populations. We gave preference for inclusion to recent studies examining the general population, but again, where there was a dearth of data, studies of subgroups of populations were included. Studies specifically relating to cancer pain were excluded.

After defining a list of clinical questions (Supplementary Materials, Appendix 2) to address the basic issues related to chronic pain (e.g., prevalence and incidence, patient demographics, comorbidity, treatment adequacy, compliance and satisfaction with treatment, or impact on quality of life), two authors independently inspected full papers of the titles and abstracts retrieved from the searches for eligibility and determined their relevance. Papers for each included country were assessed separately. For Denmark, originally, 32 included studies were categorised to obtain a list of relevant studies per question, and from these we selected for each question the most pertinent 3 papers using the following criteria: sample size and representativeness of the general population, recency, and methodological quality. For Sweden, originally 31 included papers were categorised in the same way. Overall, 11 papers were used for our Denmark review/results and 22 papers were selected to answer research questions for Sweden, some of the papers from both countries contained data for multiple questions.

We developed quality assessment criteria by utilising 9 assessment criteria formulated in accordance with the principles of STROBE checklists on reporting guidelines for observational studies [16] (Supplementary Materials, Appendix 3). For each country, one author assessed the methodological quality of each included study and a second independently checked this assessment for accuracy. Disagreements were resolved by consensus. We used this assessment for descriptive purposes allowing a transparent evaluation of the overall literature quality, based on bias risk assessment. A study was assessed as low risk of bias if the authors met all the criteria or missed only one criterion, medium risk of bias if they missed two or three criteria and high risk of bias if they missed four or more criteria.

Despite our inclusion criteria, we did not find many studies for either country with 100% inclusion of patients with noncancer pain as sometimes this criterion was unclearly reported (Supplementary Materials, Appendix 4). Therefore, we employed the terminology: “general chronic pain” that included reporting some patients with cancer pain and unclear pain populations, that is, where the population was not guaranteed to be cancer free. In addition we aimed to include only moderate to severe chronic pain. However, this was not always clearly reported and for those studies where this was not clear we used the term “any chronic pain”; these studies included patients with mild pain.

## 3. Results

**Figure 1:**
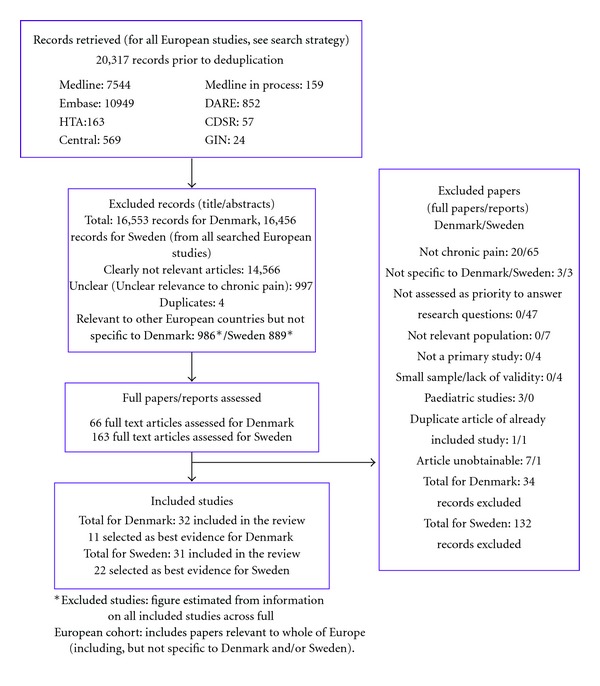
Study inclusion flow chart.

From the 20,317 references retrieved from the searches (16,619 following deduplication), we identified 66 full text articles providing potential relevant information about chronic pain for Denmark and 163 full text articles for Sweden: these were mutually exclusive for the two countries but not mutually exclusive for the research questions (references can be supplied on request, see Flow Chart in Figure 1).

For the Denmark review, no studies were identified that specifically reported the percentage of Danish chronic pain patients that are untreated or inadequately treated, the compliance of treated chronic pain patients in Denmark, levels of pain severity, the impact of chronic pain on isolation and helplessness, and comorbidities of people with pain. From 66 identified publications, 32 papers were deemed to have relevant data, but eleven primary studies were selected as the most relevant (i.e., able to elucidate specific answers to the review outcomes), reliable (i.e., robust in quality assessment), and up-to-date at the time of the review.

For the review of chronic pain in Sweden, from the full text articles, we identified 31 primary studies (at least three studies per question) that provided the most recent, representative, and valid data for Sweden; of which 22 papers were selected as best evidence for Sweden. Details of the included studies, study characteristics, and outcomes extracted for both countries are provided in Table 1, and quality assessment details in Supplementary Materials, Appendices 3 (method of assessment), and 4 (quality appraisal decisions).

In the 11 included papers selected for Denmark; four studies were judged as having low risk of bias, three of which with representative results [17–20]. For the remaining seven, we judged five to be at medium risk of bias (i.e., 2-3 quality criteria not met) [21–25] and two as high risk of bias (i.e., 4 or more criteria not met) [2, 26]. The representativeness of the results from these seven studies was assessed as unclear.

 Of the 22 selected studies for Sweden, six [29, 31, 34, 35, 42, 48] were judged as low risk of bias with results representative of the target population For the remaining 16, we judged eight to have a medium risk of bias [27, 28, 32, 38–40, 45, 47] and eight as high risk of bias [2, 30, 33, 36, 37, 41, 43, 44, 46]. (Study [33] gives additional patient information on the cohort in Study [2] so these were classed as one study.) Of the medium risk of bias studies, six were assessed as representative, one unclearly representative, and one not representative (see Supplementary Materials, Appendices 3 and 4). The main reasons for a medium or high risk of bias assessment were inadequate reporting of participant eligibility and statistical methodology. Papers were assessed individually and without reference/comparison to other papers in the review which may have reported on the same or similar populations but with different subjective descriptions of methodologies of how representativeness was assessed.

The full review assessed outcomes of chronic pain by utilising 21 clinical questions (Supplementary Materials, Appendix 2) formulating results of which only the main outcomes are presented here.

### 3.1. Demographics of Chronic Pain

In a European cohort which included a sample of adults from different areas of Denmark with moderate to severe pain, mean age for Denmark was 50.3 years and 57% were female. Pain sufferers were on average older in Scandinavian countries than other countries like Israel, Poland, and Italy; however, the sample did not include the elderly population in nursing homes [2]. In a sample of patients referred to a multidisciplinary clinic in Copenhagen [26], mean age was 49 years (SD 13) yrs with 66% women; and in another disciplinary pain centre in Funen County, Denmark [24], mean age was 48.1 (SD 13.74) years, and 61% of the sample were female.

In a sample of adult participants from different regions of Sweden with moderate to severe pain, mean age was 51.5 years, and 54% were female [2, 3]. Most came from the North of Sweden (23.2%). In a breakdown of age ranges, more people were represented in the 51–60 yrs age group (21%), followed by 31–40 yrs (20%) and 41–50 yrs (15%) [33]. In a random sample of the Swedish population, 54% of people aged 25–74 years reported suffering of various degrees of chronic pain, and 25% of the total sample complained of “high intensity” pain [27]. The median age was 51 years for people in a high-intensity group who sought primary health care (PHC) and 49.5 for those not visiting PHC. Only a small proportion of each group had been educated for >12 years and unemployment ranged from 12.6–14.6%.

### 3.2. Prevalence of Chronic Pain

For Denmark, data were obtained about the prevalence of noncancer chronic pain from three of the included studies [2, 20, 21]. From 2000 to 2005, the prevalence of chronic noncancer pain was regarded as high and stable; between 15.7% [21] and 16% [2] suffered from moderate to severe chronic noncancer pain and 20.2% [20] suffered from any chronic noncancer pain, including mild pain. When projected to adult (aged 18 and over) population figures from early 2010 [14], these frequencies represent between 691,000 (16%) and 864,000 (20%) people, respectively, in an adult population of 4.32 million. In comparison, we obtained data about the prevalence of chronic pain in Sweden from eight sources [2, 3, 27, 31, 33, 38, 42, 48]. A moderate to severe chronic noncancer pain sample reported a prevalence estimate of 18% [2, 3] for Swedish respondents. For the prevalence of any general chronic pain in discrete geographical areas of Sweden, a prevalence rate of 54.7% [27] was reported and for chronic musculoskeletal pain, prevalence was 34.5% [31]. When projected to population estimates of people aged 15 and over in Sweden in early 2010 these represent 1.40 million (M), 4.26 M and 2.68 M people, respectively [15]. Further prevalence details of individual pain populations can be perused in Table 1.

### 3.3. Impact on Quality of Life, Including Activities of Daily Living and Mental Health

For Denmark, we selected three studies [19, 20, 22], two of which were representative of the Danish population, reporting a considerable impact of chronic pain on aspects of quality of life. For Sweden, fourteen studies [2, 27, 29, 32–35, 38, 40–44, 47] were selected with data addressing various aspects of this question all reporting a considerable impact of chronic pain on aspects of quality of life, including activities of daily living, depression, and isolation; these studies ranged from low to high risk of bias (see Table 1 and Supplementary Materials, Appendix 4).

One Danish study reported that 45% of those with chronic noncancer pain rated their health as really good/good and 55% rated their health as fair/bad/very bad [22]. In contrast, 88% of the control group who reported no pain rated their health as really good/good. Another study also reported self-rated health for chronic noncancer pain sufferers [20] by utilising the 2000 chronic pain population data reported by Eriksen et al. [22] as well as data from the 2005 Danish National Health Interview Survey. The authors reported that 79.4% of those who rated their present health as very bad reported chronic pain, whereas 7.2% who rated their health as very good reported chronic pain. Both studies reported that those with chronic noncancer pain scored lower on all relevant SF-36 subscales (i.e., general health, physical functioning, role emotional, role physical, social function, vitality, and mental health) compared to those without pain [20, 22]. Those chronic noncancer pain sufferers who took opioids scored lower than those who did not take opioids.

Health related disability was measured by how physically active pain sufferers were during their leisure time [22]. Chronic pain sufferers who took opioids were significantly less active than those that did not take opioids (adjusted OR 1.55, 95% CI 1.11, 2.15) [22]. When results were adjusted for bodily pain in the last four weeks, this result was no longer statistically significant.

One study reported that significantly more sufferers of moderate to severe chronic noncancer pain stated their activities were restricted for more than six months compared to those without pain (adjusted OR 21.9, 95% CI 13.86, 34.6) [19].

Fewer (48.7%, 95% CI 44.6, 52.8) moderate to severe chronic noncancer pain sufferers reported their health as good compared to those with mild pain (80.7%, 95% CI 78.8, 82.5) or no pain (92.5%, 95% CI 91.1, 93.6). Those with moderate to severe noncancer pain scored lower on all relevant SF-36 subscales compared to those with mild pain and those without pain [19].

In Sweden, people with chronic pain reported reduced abilities for activities of daily living (ADL) [27] perceived reduced activity limitation (i.e., a perceived reduction in activities) and/or participation [43] and poor general health leading to a prevention of daily activities [42]. Lower physical functioning scores in comparison to groups without chronic pain were also reported [34]. However, in two studies, physical functioning (where a higher score indicates being able to perform more physical activities) scored more highly than bodily pain (where the highest score indicates least pain), general health (where the highest score indicates general health as excellent), and vitality (where the highest score indicates more vitality/energy) in SF-36 quality of life subscales [29, 32]. A significant proportion of people with moderate to severe chronic pain reported pain affecting their ability to concentrate and function normally and reported feeling tired all the time [2, 33].

Many Swedish participants with pain reported levels of depression and/or anxiety and related symptoms like insomnia [2, 33, 34, 42]. Elevated levels of feeling alone, isolated, and helpless and reduced levels of social contact as well as dissatisfaction with social contacts were also reported [2, 33, 44, 47]. Participants with chronic spinal pain expressed a range of responses for social support on the multidimensional pain inventory (MPI) [35].

### 3.4. Economic Impact of Chronic Pain on Individuals, Society, and Healthcare Utilisation

For Denmark, we selected six studies, all with unclear representativeness but varying risks of bias, suggesting that chronic pain has an economic impact on individuals, society, and healthcare utilisation [2, 18, 22–24, 26]. For Sweden, ten studies to elucidate the economic impact of pain on various outcomes and factors [2, 3, 30, 34, 38, 40, 44–47], risk of bias varied from low to high (see Table 1 and Supplementary Materials, Appendix 4).

In Denmark, the mean number of work days lost due to moderate to severe chronic noncancer pain per respondent in the last six months was 9.4 days [2]. In those with general chronic pain, respondents reported a mean of 0.8 days of illness in a 14-day period, and persons with pain were at increased risk of quitting their job (OR 7.3 (95% CI 6.2, 8.6) and at increased risk of absence due to illness (OR 2.0, 95% CI 1.6, 2.4) [18]. Opioid users had increased risk of having disability pension (OR 2.03, 95% CI 1.38, 2,99) and decreased risk of being engaged in employment (OR 0.45, 95% CI 0.31, 0.65) [22]. Alternatively, in one large Swedish cohort, 40.6% had a period of sick leave <3 months and 15.1% reported being off sick for >3 months; the mean period of sick leave was 43 days [44]. In other, smaller studies 69% of people with chronic pain was reported to be on sickness benefit [47], and another sample reported just 22% of the sample being employed or studying, with 26% taking early retirement or disability pension [30]. In adults with moderate to severe chronic pain, 37.6% of the sample reported to be in employment. Of the total sample 24% reported losing their job due to issues arising from their pain, 28% had changed job responsibilities, and 25% had changed jobs entirely [2, 3].

29% of Swedish participants with chronic neck pain reported being off sick due to their pain [38], and in a cohort of patients with fibromyalgia (FM) pain 37.1% were reported to be on sickness benefits compared to 12% of patients with non-FM pain [34]. In another sample of FM patients [46], 55% of people was drawing a sickness pension.

Costs of chronic pain in Denmark were reported in three studies. One study estimated the mean total costs of healthcare in patients applying for disability pension because of chronic noncancer pain at €4419 (Danish Krone (DKK) 33,139) per year [23]. Costs of secondary health care accounted for 94–98% of total healthcare costs with no differences between men and women. Another study of patients referred by a GP to a pain clinic in a hospital setting reported healthcare costs only in terms of regression analyses without total costs. In the regression analyses annual healthcare costs increased with age by about €4,200–€6050 (DKK560-806) per person per year [24]. The annual healthcare costs in the year prior to pain onset were €1160 (DKK 8,699) per person higher than in the previous years. Mean costs of council services per patient per year were €2008 (DKK15, 060) of which €1662 (DKK12, 468) was used for personal care and €346 (DKK2, 592) for housekeeping, gardening and other services. Total costs for the patient were €2051 (DKK 15,386) per patient per year [24]. Another study reported the change in health care resources and social transfers as a consequence of multidisciplinary pain treatment in a group of chronic noncancer pain sufferers [26]. Total costs were estimated at €7707 (DKK57, 802) for a period of 29 months including the multidisciplinary pain treatment. Cost of social transfers (welfare benefit, sickness benefit, and pensions) was estimated at €9514 (DKK71, 355) for the whole period of 29 months.

We located only two studies which reported on monetary costs of chronic pain in Sweden. In people with chronic back pain total average annual direct cost per patient was (Euros) €3089 (15% of total costs), but when indirect costs were taken into account, total annual costs per patient were estimated at €20,666 [36]. In patients with RA, the mean annual total cost per patient was 108,370 SEK (Swedish Kronor) (€12,286) with annual direct costs of 44,485 SEK (€5,043) (41% of the total costs). Costs to individual patients were estimated at 4302 SEK (€488), and this included the cost of informal care [40]. Neither of these studies was clearly representative of target populations. Conversion rates of 1 Euro (€) = 7.50 Danish Krone (DKK) and 1 Euro (€) = 8.82 Swedish Kronor (SEK) were used by the reviewers to present results in equal measures [49].

### 3.5. Chronic Pain Presentation, Treatment Pain Control, and Satisfaction with Treatment

We selected seven studies from Denmark, of which two were representative, which reported on presentation, treatment and pain control, with treatment [2, 3, 17, 20–23] (Table 1 and Supplementary Materials, Appendix 4). For Sweden we selected five studies which reported on presentation, treatment and pain control and satisfaction with treatment of chronic pain in Sweden [2, 27, 33, 37, 44], although some studies had a high risk of bias and were unclearly representative (see Table 1 and Supplementary Materials, Appendix 4).

In Denmark, of those with moderate to severe chronic noncancer pain, 14% had seen a pain management specialist at least once [2]. Forty-seven percent was currently taking prescription medication. Of these, 74% reported inadequate pain control. In Sweden, of those with moderate to severe chronic noncancer pain [2], 35% reported not having their pain treated in any way. 46% was taking prescription medication; 36% had tried prescription medication but then stopped. 12% reported seeing a pain management specialist when asked the question specifically, and the majority of treatment was given by general practitioners (69%). 17% of participants had obtained alternative care for their pain symptoms [3].

Of those with general chronic noncancer pain in Denmark, the proportion who had contact with a medical doctor within the last 3 months varied between 59% and 78% [22, 25]. The mean number of visits to GP in a group of individuals who claimed compensation for their pain was approximately 8 per year [23]. The proportion that was satisfied with medical treatment was 52% among opioid users and 56% among nonopioid users and inadequate pain control was reported to be twice as high among opioid users compared to nonopioid users [22]. In 2005, 45.9% of chronic pain patients in Denmark were not satisfied with the pain treatment they received [20]. In Sweden, in participants with any general chronic pain in the three months prior to the study, when compared to participants without chronic pain, significantly more people consulted physicians, physiotherapists, and primary health care (PHC) doctors (45.7% versus 29.8%, 7.2% versus 1.2%, and 39.5% versus 25.5%, resp.) [27]. 12.3% of people with chronic pain made at least one visit to hospital clinics in the last three months compared with 7.3% without pain. Only 5.9% of people with chronic pain used alternative care, but 58.2% of people with chronic pain had taken their own steps to reduce the pain and if self-medication was included, 73.1% had performed some activity to reduce pain. Patients with chronic pain were most likely to consult physicians, physiotherapists, chiropractors, nurses, and occupational therapists for their treatment [44]. In another study with a sample of adults with general chronic pain [37], 64.8% of people sampled were reported as seeking health care for their pain. Of these, 86.3% was in constant pain, and 79.5% reported severe pain. In a sample of patients in Sweden with chronic low-back pain (CLBP) patients scored a median of 3 on a 6-point scale, indicating they were somewhat dissatisfied with their pain relief, tolerance, and overall treatment [36].

### 3.6. Frequency of Pain Treatments

Four Denmark studies reported on the frequency of various pain treatments [2, 17, 22, 24] with varying risks of bias and representativeness. For Sweden, four studies (across three cohorts) were utilised to extract the frequency of pain treatments [2, 27, 33, 45]. All studies had a medium or high risk of bias. 

In Denmark, amongst a group of moderate to severe pain sufferers, the proportion of patients who were prescribed WHO class I, II, or III drugs was 46%, 8% and 11% respectively [2]. Other studies in our paper reported that 11.2% and 12% used opioids [17, 22]. The most frequent nondrug pain treatment tried by those with moderate to severe general chronic pain was physical therapy (23%) followed by massage and acupuncture (each 21%) [2]. Of patients on a waiting list for treatment at a pain clinic, 79% received alternative treatments, such as acupuncture (43%), massage/manipulation (42%), and reflexology (31%) [24]. Around 16% of opioid users and 18% of nonopioid users had tried massage, osteopathy, or another manipulative therapy [17].

In Sweden, The most frequent WHO class drug treatments prescribed to patients with moderate to severe chronic pain in Sweden were paracetamol (26%), NSAIDs (27%) and Step II Opioids (36%), and nonprescription drugs taken were paracetamol (75%) and NSAIDS (25%) [2, 33]. In another paper visitors to healthcare were more likely than nonvisitors to healthcare to use both prescription and nonprescription drugs (59.6% versus 35.6% and 62.2% versus 48.3% resp.) [27].

The most frequent nondrug pain treatments tried by those with moderate to severe general chronic pain were physical therapy (55%) acupuncture (41%) and massage (36%) [2]. In patients with moderate to severe pain (from a larger cohort including people with milder pain), nonvisitors to healthcare had only slightly higher levels of alternative treatments compared to people visiting healthcare (10.3% versus 8.9%). In the full cohort, only 5.9% of participants had accessed alternative treatments in the last 3 months [27].

In a sample of patients with chronic pain due to spinal cord injury (SCI) [45] 70.5% were reported as having tried either drug or nondrug treatments, and 51% were using drugs at the time of the study. 41.2% of the sample had tried one or more analgesics. People with moderate pain, adjusted OR 4.94 (95% CI 1.5, 16.7) or severe pain, adjusted OR 10.45 (95% CI 2.0, 54.7) on the VAS scale were more likely to try nondrug therapy than people with mild pain only. 34% of participants was reported as using opiate analgesia and the most popular nondrug therapies were acupuncture (35.6%) and massage (34.4%).

### 3.7. Severity and Duration of Pain

Only one paper from Denmark reported on duration of chronic pain [2], and no specific data on severity was located. We selected four papers that best reported on the severity and duration of chronic pain in Sweden, with varying degrees of bias [2, 3, 35, 40].

The mean duration of pain in years in moderate to severe chronic pain was 8.3 for Denmark and 9 for Sweden [2]. In Sweden, 13% of people had used pain intensity scales before to evaluate their pain, and 24% of people scored as having severe pain (i.e., 8–10) on the 10-point NRS scale. 46% reported experiencing chronic pain daily, and 33% reported to be in pain all the time [3]. 36% said their pain was so severe that no more could be tolerated. In a sample of patients with chronic spinal pain 24% of people reported being in pain for between 3 and 12 months, and 76% had pain for over 12 months [35]. On a 1–100 score (100 being most pain), the mean pain score was 44.8 (SD18.6) for the <12 month group and 47.3 (SD 17.7) for the >12 month group. Finally in a sample of people with rheumatoid arthritis (RA), mean duration of disease was 16.7 years (SD12.9) and mean pain score was 40 (SD24) [40].

### 3.8. Comorbidities and Underlying Diseases of Pain Sufferers

Once again, we did not elicit any information from Demark for this outcome. We included six papers with data reporting comorbidities and underlying diseases for people in Sweden experiencing chronic pain, and once again the risk of bias and degree of representativeness assessed was varied [2, 3, 28, 35, 39, 42], see Supplementary Materials, Appendix 4.

In Swedish papers with data relating specifically to comorbidities, 32% of a sample with chronic upper extremity pain also experienced chronic upper extremity numbness or tingling. 20.8% of participants had a physical impairment alongside chronic pain [39]. Among those younger than 65 years, many comorbidities were seen to exist with chronic widespread pain (CWP) with the most likely being chronic fatigue OR 3.71 (95% CI: 2.06–6.70), joint pain OR 4.60 (95% CI: 2.63–8.04), and possible rheumatoid arthritis (RA), OR 3.89 (95% CI: 1.87–8.09). Headaches and depression were also commonly found [42]. Those with chronic nonspecific spinal pain had a significantly higher hospital anxiety and depression (HADS-D) mean score than those with recurrent nonspecific pain lasting <3 months (4.2 [SE 0.24]; *P* < 0.05) [35].

The remaining studies reported on underlying disease linked to chronic pain. In chronic musculoskeletal pain (CMP), the most prevalent underlying diseases in participants were back pain, 36.7 per 1000, fibrositis-myalgia, 33.0 per 1000, and local tendinitis-bursitis, 28.6 per 1000 [28]. In moderate to severe pain the most common underlying health problems were arthritis/OA (27%); traumatic injury (19%) and nerve damage (17%) [2, 3].

## 4. Discussion

This paper was written as part of a large Europe wide project exploring the epidemiology of chronic pain in literature reviews across several discrete European countries and across Europe as a whole [9–11].

 We conducted separate comprehensive epidemiological literature reviews in the broad field of chronic pain, its impact, and treatment, and we present here our methods and results for Denmark and Sweden. Overall, we assessed that although there were a number of papers elucidating data on chronic pain in both countries, our inclusion criteria of papers with noncancer chronic pain, clear representativeness to the general population, and a low risk of bias were sometimes difficult to achieve. Due to unclear reporting, we judged the validity or representativeness of the data from eight of eleven Denmark studies as unclear: only four studies were judged as low risk of bias. For Sweden, we judged the validity or representativeness of the data as having low risk of bias combined with high representativeness in just six of the 22 included papers (Supplementary Materials, Appendices 3 and 4). Only one source (with two papers reporting on both countries), reported broadly on chronic noncancer pain where less than 1% of the study population reported their pain as cancer related [2, 3].

The prevalence of moderate to severe chronic pain in Denmark is estimated at 16% for moderate to severe noncancer pain [2] and at 20.2% for any chronic noncancer pain [21]; the estimate for moderate to severe noncancer pain in Sweden is similar at 18% [2]. This is comparable with recent estimates for the Netherlands 18% [9], Germany 17% [11], and across Europe as a whole at 19% [10]. In a recent unpublished review, estimates for non-malignant chronic pain prevalence for Italy were 26%, 12-month prevalence of moderate to very severe general non-cancer chronic pain in an alternative sample from Denmark [21] was 15.7%. In contrast, Swedish studies assessing prevalence of general chronic pain in a discrete geographical area of Sweden reported a much higher prevalence rate of 54.7% [27].

In comparison with other chronic diseases, the overall prevalence of Type 2 diabetes mellitus in Denmark was estimated at 4% in 2007 [50] and 3.03 cases per 1000 in Sweden [51]. The prevalence of major depression was 3.3% in Denmark [52] and between 4–8% in Scandinavia as a whole [53]. The mean annual coronary event rates in males and females aged 35–64 were 517 and 140, respectively, per 100,000 in Denmark and 509 and 119, respectively per 100,000 in Northern Sweden [54]. These data indicate that in comparison with other life-affecting diseases, chronic pain is an important public health issue in both Denmark and Sweden.

The review data from both countries reported various evidence that chronic pain in both Denmark and Sweden has a clearly negative impact on quality of life, activities of daily living, physical functioning, and general wellbeing for those affected. Chronic pain in many capacities was associated with depression and anxiety, isolation and helplessness, increased sick leave, and increased uptake of benefits and pension.

Although no formal economic evaluations of chronic pain in Denmark or Sweden were found, results from several studies support the statement that chronic pain has an economic impact on society, health care resources, and the individual. Around 9 workdays per 6 months are lost due to chronic pain in Denmark, and there was an association between chronic pain and disability pensions, suggestive of economic impact of pain on society [2, 19, 22]. In one large Swedish cohort, 40.6% had a period of sick leave under 3 months and 15.1% reported being off sick for over 3 months; the mean period of sick leave was 43 days [44]. These figures have implications in social and benefits costs for both countries. Total costs of health care were however difficult to interpret as studies referred to different patient groups and different study periods. Broader population economic evaluation studies are needed to enlighten healthcare providers and researchers on the economic burden of chronic pain in each country.

There is evidence that chronic pain sufferers in Denmark and Sweden are undertreated. In Denmark, between 59% and 78% of chronic pain sufferers had contact with a medical doctor in the last three months [22, 25], and only 19% of sufferers of any chronic pain and 14% of those with moderate to severe chronic pain had ever seen a pain management specialist [2, 23]. About three quarters of those with moderate to severe chronic pain report inadequate pain control with prescription pain medication [2], and 90% of opioid users for general chronic pain reported inadequate pain control [22]. About half of chronic pain patients were unsatisfied with the treatment they received [20]. In our reviews, we interpreted medications as being any substance taken to combat pain, for example analgesia as described by the International Association for the Study of Pain (IASP) [55] either prescribed by a doctor or bought over the counter in a pharmacy or elsewhere. We also collected information about other types of prescribed medication (e.g., antidepressants, anticonvulsants) which were recorded as being prescribed to combat pain. Alternative treatments were interpreted and/or reported as offering solutions other than medication to treat pain (e.g., physical treatments like massage, osteopathy, and swimming).

In Sweden, although the majority of people with chronic pain in the studies reported seeking treatment of either conventional medical care or alternative care for their chronic pain, a significant minority (35.2% in one sample [37]) reported not seeking/receiving treatment. In another study only 12% of Swedish patients with moderate to severe chronic pain reported seeing a pain management specialist [2]. This gives cause for concern and has implications for the provision of chronic pain care and facilities in Sweden. Very little data (and only on discrete populations) were found on patient satisfaction in Sweden, but the small amount we found indicated low levels of treatment satisfaction; no papers or data on compliance were located.

In our reviews, we did not set out to find information regarding detail on which specific treatment modalities were deemed to be insufficient either by patients or doctors and the possible reasons for this, although this should not be ignored in future research, both primary and secondary. However it is noted that 79% of patients attending a multidisciplinary pain clinic in Denmark [24] received “alternative treatments”. In a previous randomised trial of chronic nonmalignant pain patients managed in a Danish multidisciplinary pain centre compared to general practice [56], better pain outcomes were found in the multidisciplinary centre, which the authors found may be due to increased used of nonanalgesic medications such as antidepressants and anticonvulsants; this has implications for further research and treatment of individuals with chronic pain. No information was found on these types of pain clinics in Sweden.

Although our literature reviews were conducted using the highest quality data available, some limitations must be acknowledged. The reviews were performed within limited time constraints and budget and were not designed to be full systematic reviews. Notwithstanding this, thorough literature reviews containing the most relevant assessed information and data, extracted and checked by two reviewers at all stages were carried out. The authors assessed many of the individual studies as having various limitations in their reporting of statistical methods and effect sizes, and in some studies, only descriptive statistics were reported. It is well documented that reporting of such research can be inadequate, and that this may make it difficult to assess generalisability of observational studies [57].

We also noted that many of the selected published studies for both countries were written by the same authors, often intermixed with other frequently cited authors for chronic pain; in our Denmark review, two of the authors were cited repeatedly (with other authors) in nine of our selected studies (see references); this phenomenon was less marked in our Swedish review but never-the-less there were some sets of authors writing papers on the same pain populations within similar time frames in different journals; for example, [27, 28, 43, 44]. Although it is commendable for clinicians and researchers to report and publish their data; it must also be noted that when authors are involved in multiple pain populations there is potential for study bias if there is a lack of clear and transparent reporting on study populations, outcomes, and any similarities in methods and within or between populations, in each study that is published [57]. There is also potential for duplicate publication bias if authors publish data on the same populations in different publications without making this obvious; this can lead to a misinterpretation or overanalysis of data [58].

The authors of this paper made judgements on risk of bias and representativeness according to a quality assessment checklist (Supplementary Materials, Appendix 3) formulated in accordance with the principles of STROBE checklists on reporting guidelines for observational studies [16, 57, 59]. In a few cases our judgements were not fully compatible with judgements made by authors of individual studies, where these were reported, highlighting the subjectivity of reporting methodological quality in observational studies of this type.

## 5. Conclusions

Chronic pain is a common and important public health problem in both Denmark and Sweden. It impacts all aspects of quality of life negatively and probably also has a negative economic impact on society, health care resources, and the individual. The results of both reviews suggest that chronic pain sufferers are undertreated and that more reliable epidemiological data are needed to fully define the burden of chronic pain in these two Scandinavian countries. In order for future treatment and management of chronic pain to be optimal in Denmark and Sweden, research should contain high-quality, relevant statistical information and be representative of relevant populations.

## Supplementary Material

Appendix 1 is the full Medline Strategy we utilised for the searches. This strategy was adapted specifically for each subsequent database and the keywords associated with epidemiology and chronic pain were adapted according to the thesaurus and configuration of each individual database. Appendix 2 is a list of clinical questions that were addressed systematically in our literature review. The reviews were initially conducted separately with each question being applied for each country. Where results are not presented for a particular question, this is because either (a) no papers were found addressing the question or (b) information/data found was not considered to be of appropriate study quality or relevance to the review. Appendix 3 describes the detailed quality assessment criteria which we used to assess each included study. All studies were assessed on all eight criteria/questions, and finally a score was generated (see section 9) to assign a risk of bias for each study i.e. low, medium or high risk. Appendix 4 contains the results of the quality assessment for each included study from Denmark and Sweden. The parameters 1-8 reflect the answers/scoring to Questions 1-8 on the quality assessment measure (see Appendix 3). The final column shows the risk of bias assessed for each individual study.

## Figures and Tables

**Table 1:** Summary of all papers included in the Denmark (*n* = 11) and Sweden (*n* = 22) literature reviews. Risk of bias and study representativeness, study characteristics, and results of relevant outcomes extracted.

Paper and reference number, country	Number of participants, demographic data (age and gender if reported), and target population	Type of study/study quality/risk of bias and representativeness	Chronic pain condition and main study outcomes reported
Breivik et al. 2006 [2]/Fricker Pain in Europe (PIE) 2003 [3]* Multicountry study—data taken for Denmark and Sweden	*Denmark * *n* = 2169 for prevalence outcome, *n* = 303 for all other outcomes (interviewees from Denmark); mean age 50.3 years; 43% men *Sweden * *n* = 2563 for prevalence outcome *n* = 300 for all other outcomes (interviewees from Sweden) Mean age 51.5 yrs, 46% women *Target population * Adult general population ≥18 years	Observational survey High risk of bias Representativeness unclear	*Chronic pain condition * Moderate to severe chronic noncancer pain lasting ≥6 months (1% reported cancer-related pain) *Outcomes reported (Denmark/Sweden) * Prevalence: 16%/18% *Mean duration of pain in years:* 8.3/9.0 Economic impact: *Work days lost in the past 6 months: *mean 9.4 days/mean: 7 days *Number of people in the sample reported as employed: *135/303 (45%) /113/300 (37.6%) *Proportion who felt that pain impacted upon employment.* Due to pain: 29%/24%, lost their job, 11%/25% changed job, and 21%/28% changed job responsibilities Presentation and treatment of chronic pain: (1)* Proportion who took prescription medication:* 47%/65% (2)* Proportion with inadequate pain control from medication: *74%/30% (3)* Proportion with inadequate pain control overall: 61%/45% * (4)* Proportion of people reported seeing a “pain management specialist”: 14%/12% * Frequency of prescribed treatments *Drug treatment:* NSAID 38%/27%, weak opioid 8%/36%. Strong opioid 11%/3%, paracetamol 0%/26%, COX-2 inhibitor 8%/7% *Most common nondrug treatments *massage: 21%/36%, Physical therapy: 23%/55%, acupuncture 21/41%

Ekholm et al. 2009 [17], Denmark	*n* = 5292 (4115 had no chronic pain); 46.3% men *Target population * General population	Observational survey Low risk of bias Representative	*Chronic pain condition * General noncancer chronic pain (≥6 months) *Outcomes reported * Frequency of treatments *Drug treatment: *opioid usage rate: 11.2% *Nondrug treatment:* approx. 16% of opioid users and 18% of nonopioid users had tried massage, osteopathy or another manipulative therapy

Eriksen et al. 2003 [18], Denmark	*n* = 10 066 (pain group: *n* = 1871, control group (general population sample without pain): *n* = 8195); 48% men *Target population * Adult general population ≥16 years	Observational survey Low risk of bias Representativeness unclear	*Chronic pain condition * General chronic pain lasting six months or more *Outcomes reported * * Work loss days lost in past 6 months: *mean: 9.6 days (versus 4.8 in control group) *Odds ratio of quitting job (pain group versus control):* OR = 7.3 (95% CI: 6.2, 8.6) *Odds ratio of absence due to illness (pain group versus control): *OR = 2.0 (95% CI: 1.6, 2.4)

Eriksen et al. 2004 EJP [25], Denmark	*n* = in 2000, 10 066 (1906 with pain); 48% men, control group (no pain) *N* not reported. *Target population * Adult general population ≥16 years	Observational survey Medium risk of bias Representativeness unclear	*Chronic pain condition * 1994 survey: VRS 4, 5, or 6 (moderate, severe, or very severe pain), 2000 survey: pain lasting 6 months or more' Cancer patients were excluded. *Outcomes reported * Presentation and treatment of chronic pain *Proportion of patients who had at least 1 consultation to a medical doctor within the past three months (in 2000):* 59–78% *Proportion who had contact with specialists: *19%; most commonly surgeons (4.1%) and rheumatologists (4.3%)

Eriksen et al. 2004 Pain [21], Denmark	*n* = 2649 (in 2000, 357 with chronic pain); 47% men *Target population * Adult general population ≥18 years	Retrospective analysis Medium risk of bias Representativeness unclear	*Chronic pain condition * Moderate to very severe general noncancer chronic pain (>6 months) *Outcomes reported * Prevalence: in adults ≥18 yrs: 12-month prevalence in 2000:15.7%

Eriksen et al. 2006 [22], Denmark	*n* = 10 066 (1906 with pain); 42% men. Control group (patients not reporting chronic pain) *n* = 8,106, 50% men *Target population * Adult general population ≥16 years	Observational survey Medium risk of bias Representativeness unclear	*Chronic pain condition * Chronic noncancer pain lasting ≥6 months *Outcomes reported * Impact of chronic pain *General health. Self-rated health status (5 point scale): *45% of those with chronic noncancer pain rated their health as really good/good and 55% rated their health as fair/bad/very bad. In contrast, 88% of the control group who reported no pain rated their health as really good/good. *Age adjusted mean scores for SF-36 subscales (general health, physical function, role emotional, role physical, social function, vitality, mental health).* Those with chronic noncancer pain scored lower on all relevant SF-36 subscales compared to those without pain. No statistical analyses were reported. Chronic pain sufferers who took opioids were significantly less active than those that did not take opioids (adjusted OR 1.55, 95% CI 1.11, 2.15) Economic impact *Odds ratio of opioid use (versus nonopioid use) and not being engaged in employment: *OR = 0.45 (95% CI: 0.31, 0.65) *Odds ratio of opioid use (versus nonopioid use) and having disability pension: *OR = 2.03 (95% CI: 1.38, 2.99) Presentation and treatment *Proportion who had contact with a medical doctor within the last 3 months: *69.8% *Proportion inadequate pain control: *opioid users 90%, nonopioid users 46% *Proportion satisfaction with medical treatment:* opioid users 52%, nonopioid users 56% *Prescribed treatments; analgesics by type* Analgesics of any type: 30%, opioids 12% (9% weak and 3% strong), anxiolytics 3%, antidepressants 4%

Højsted et al. 1999 [23], Denmark	*n* = 144; median age 51 years, 33% men *Target population * Patients of Danish origin with chronic nonmalignant pain and applying for a disability pension due to chronic pain	Retrospective cohort study Medium risk of bias Representativeness unclear	*Chronic pain condition * Nonmalignant “chronic pain” (not further defined) *Outcomes reported * Economic impact *Proportion of chronic noncancer pain patients that were finally denied a disability pension: *44.45% *Proportion of chronic noncancer pain patients that were finally awarded a disability pension:* 55.55% *Mean total costs of healthcare in patients applying for disability pension: *33 139 DKK per year, (=Euro €4449) Presentation and treatment *Mean number of visits to GP in a group of individuals with chronic noncancer pain who claimed compensation for disability:* 8 in the year before claim and 7.7 in the year following the final decision *Mean number of visits to outpatient clinics: *1.7 and 1.2, respectively

Jensen et al. 2004 [19] Denmark	*n* = 3992 (*n* = 563 high pain group (HPG); *n* = 1715 low pain group (LPG); *n* = 1714 control group (CG) i.e., no pain); 52.2% men *Target population * Adult general population	Observational survey Low risk of bias Representative	*Chronic pain condition * Moderate to severe general noncancer chronic pain. Chronic was not defined *Outcomes reported * Impact of chronic pain *General health. Self-rated health status (5 point scale):* fewer (48.7%, 95% CI 44.6, 52.8) moderate to severe chronic noncancer pain sufferers reported their health as good compared to those with mild pain (80.7%, 95% CI 78.8, 82.5) or no pain (92.5%, 95% CI 91.1, 93.6) *Adjusted mean scores for SF-36 subscales (general health, physical function, role emotional, role physical, social function, vitality, mental health).* Those with moderate to severe noncancer pain scored lower on all relevant SF-36 subscales compared to those with mild pain and those without pain. No statistical analyses were reported. *Health-related disability (long-lasting activity restriction [>6 months] due to ill health). *More sufferers of moderate to severe chronic noncancer pain stated their activities were restricted for more than six months compared to those without pain (adjusted OR 21.9, 95% CI 13.86, 34.6)

Kronborg et al. 2009 [24], Denmark	*n* = 204; mean age 48.1 (SD 13.74) years; 39% men *Target population * All patients referred to the Multidisciplinary Pain Clinic in Funen County at Odense University Hospital, Denmark and on the waiting list as at 1st December 2005.	Observational survey Medium risk of bias Representativeness unclear	*Chronic pain condition * General noncancer chronic pain *Outcomes reported * Economic impact *Percentage of work hours lost due to chronic noncancer pain: *41.0% (SD = 23.00) *Healthcare costs (by regression analysis):* costs increase with age—about DKK 560–806 (€4,200–€6,500) per person per year Costs are higher in year prior to pain onset—about DKK 8,699 (€1,159) per person compared to the previous years *Mean costs in Danish Krone (DKK) for council: * (1)* Personal care services, *(2)* housekeeping, gardening, and so forth: * (1) DKK 12,468 (€1662), (2) DKK 2,592 (€346) *Total costs in DKK:* DKK 15,060 per patient per year (=Euro (€) 2008) *Mean costs for patients: * (3)* Privately provided services, *(4)* alternative treatment:* (3) DKK 12,408 (€1654), (4) DKK 2,978 (€397) *Total costs in DKK:* DKK 15,386 per patient per year(=€2051) Frequency of treatments *Nondrug treatments (drug treatments not reported):* 79% alternative treatments: 43% acupuncture, 42% massage/manipulation, 31% reflexology

Sjøgren et al. 2009 [20], Denmark	*n* = 5292 (all with chronic pain); 47.7% men *Target population * Adult general population ≥16 years	Observational survey Low risk of bias Representative	*Chronic pain condition * General noncancer pain ≥6 months *Outcomes reported * Prevalence. In adults ≥18 yrs: 12-month prevalence in 2005: 20.2% Impact of chronic pain *General health.* Self-rated health status (5 point scale): 79.4% of those who rated their present health as very bad reported chronic pain, whereas 7.2% who rated their health as very good reported chronic pain *Age adjusted mean scores for SF-36 subscales.* Those with chronic noncancer pain scored lower on all relevant SF-36 subscales compared to those without pain. No statistical analyses were reported Presentation and treatment *Proportion of chronic pain patients not satisfied with the chronic pain treatment they received: *44.5% in 2000, 45.9% in 2005

Thomsen et al. 2002 [26], Denmark	*n* = 131; mean age 49 (SD 13) yrs; 34% men *Target population * Patients (age above 18 years) who were consecutively referred to the Multidisciplinary Pain Center at the National Hospital, Copenhagen (1995–1997)	Observational survey High risk of bias Representativeness unclear	*Chronic pain condition * General nonmalignant chronic pain lasting 6 months or more; low back pain: 32% *Outcomes reported * Economic impact *Proportion of chronic noncancer pain patients that were applying for a disability pension * (1)* At referral to pain clinic 20%, *(2)* during treatment 9%, *(3)* at followup 4% * *Total health care costs (period of 29 months including multidisciplinary pain treatment) *7707 Euro (€), (DKK 57,802), (2) *total medication costs, *€1476 *(DKK 11,070), and *(3)* total social transfers (costs of welfare benefit, sickness benefit and pensions): *€9514 (DKK 71,355) *Total social transfers per period (per patient per month): *(1)* prior to referral* €459 (DKK 3,442), (2) *waiting list* €398 (DKK 2,985), (3) *treatment period* €354 (DKK 2,655), (4) *followup period:* €172 (DKK 1,290)

Andersson et al. 1999 J Epi Comm [27], Sweden	Sampled *n* = 1806; responded *n* = 1607; *n* = 872 had any chronic pain: median age 49.5 years; 49.4% men *Target population * Adult general population aged 25–74	Observational survey Medium risk of bias Representative	*Chronic pain condition * Any general moderate to severe chronic pain *Outcomes reported * * Prevalence of general chronic pain:* 54.7% *Prevalence of chronic back pain:* 15.4% *% of high intensity pain patients with reduced capacity for Activities of Daily Living (ADL).* Total reduced ADL capacity: 13%, study compared visitors and nonvisitors to Primary Health Care (PHC): Visitors to PHC: 13.8%, Nonvisitors to PHC: 12.1% (difference between groups not significant) *Percentage consulting physician or physiotherapist in last three months: *45.7% consulted a physician (versus 29.8%, *P* < 0.05 of nonchronic pain persons) and 7.2% consulted a physiotherapist (versus 1.2%, *P* < 0.05 in nonchronic pain individuals) *Percentages of visits to Primary Health Care (PHC), Hospital Care, and Alternative Care, compared to people without chronic pain: PHC doctor*—39.5% of people with chronic pain consulted (c/w 25.5%, *P* < 0.05), people with pain of 3–6 months duration: 59% PHC contacts compared with those with pain of >6 months duration: 34%, *Hospital Care*—12.3% of people with chronic pain made at least one visit to hospital clinics in the last three months (c/w 7.3% without pain—NS). 2.8% of chronic pain individuals had an emergency contact during this period *Alternative Care*—5.9% of people with chronic pain used alternative care. Chiropractor—3.7% (*n* = 32) Acupuncture—1.7% (*n* = 15) Homeopathy, zone therapy, and other naturopathy—% NR. 58.2% took steps to relieve the pain themselves *Proportion of people taking steps to reduce pain themselves. *Visitor to PHC: *n* = 225; median age 51.0 yrs; 55.1% female. Nonvisitor to PHC: *n* = 174; median age 49.5 yrs; female 50.6% *Number of “high intensity” visitors/nonvisitors to Primary Health Care (PHC). Type of care received: *self-care: 58.2%, conventional medicine: 47.1%, alternative care: 5.9%, combined conventional and alternative care: 3.1% *Drug treatments. Visitors/nonvisitors to healthcare:* Prescriptions: 59.6%/35.6%, nonprescriptions: 62.2%, 48.3% *Alternative treatments. Visitors/Nonvisitors to Health Care:* 8.9%/10.3% *Analgesic use in the past 2 weeks: *62.4%, *alternative treatments in the last 3 months,* any: 5.9%, chiropractor: 3.7%, acupuncture: 1.7%

Andersson et al. 1999 Scand J PHC [28], Sweden	Total sampled *n* = 1101, chronic pain *n* = 534, mean age not reported, 49.6% men *Target population * Adults in Bromölla health district aged 25–74 years	Observational survey (and continued diagnosis registration) Medium risk of bias Representative	*Chronic pain condition * any chronic widespread pain; any chronic neck-shoulder pain (cancer not excluded) *Outcomes reported * * Diagnoses related to chronic musculoskeletal pain in 1996 (per 1000)* Back pain 36.7, fibrositis-myalgia 33.0, local tendinitis-bursitis 28.6, other musculoskeletal disease or symptom 25.7, arthralgia 15.1, osteoarthritis 9.0, neck syndrome 8.8, shoulder syndrome 8.0, inflammatory joint and collagen disease 7.8, total diagnoses related to musculoskeletal pain 192.6

Arvidsson et al. 2008 [29], Sweden	*n* = 2425 responders; mean age 50.3 yrs, 39% men *Target population * Adult general population of Sweden ≥18 years	Longitudinal study Low risk of bias Representative	*Chronic pain condition * Any chronic musculoskeletal pain *Outcomes reported * * Mean of SF-36 subscales (scale 0–100, higher is better health): *physical functioning 75, role-physical 60, bodily pain 50, general health 60, vitality 55, social functioning 80, role-emotional 73, mental health 73

Ben-Menachem et al. 1995 [30], Sweden	*N* = 96, mean age 55.1 yrs, 44% men *Target population * Patients with chronic pain in Sweden ≥18 years, and their spouses	Observational survey High risk of bias Representativeness unclear	*Costs to society—proportion of patients employed or on pensions and benefits.* Fully employed or in school: 22%, employed but in partial sick-leave: 14%, temporary disability pension: 8%, housewife: 4%, early retirement or disability: 26%, retirement pension: 26%, unemployed: 0%

Bergman et al. 2001 [31], Sweden	*n* = 2755 responders, *n* = 2425 analysed, mean age 46.5 yrs, 47% men *Target population * Adult population of South-West Sweden aged 20–74 yrs	Observational survey Low risk of bias Representative	*Chronic pain condition * Any chronic regional or widespread musculoskeletal (MSK) pain (cancer not excluded) *Outcomes reported * * Prevalence of any musculo-skeletal chronic pain:* 34.5% *Prevalence of chronic low back pain: women/men:* 26.4%/19% *Prevalence of chronic neck pain: women/men:* 22.9%/14.5% *Prevalence for chronic widespread Pain: *11.4% *% of people reporting pain at 8, 9 or 10 on the 10-point NRS scale: 35%/24% * *Mean of SF-36 subscales for CRP, CWP, and fibromyalgia, respectively (Scale 0–100, higher is better health): *physical funct. 80, 63, 50, role-physical 64, 45, 20, bodily pain 54, 40, 28, general health 65, 49, 33′ vitality 59, 44, 33, social functioning 84, 71, 58, role-emotional 78, 60, 45, mental health 78, 65, 58

Bergman 2005 [32] Sweden	Responders *n* = 2425. Proportion of males/females not reported–response rates were 57.5%/66% respectively Mean age not reported * Target population * Adult population of south-west Sweden aged 20–74 yrs; responders *n* = 2425, CRP *n* = 588, CRP *n* = 303, FM *n* = 15, assumed to be same population as Bergman 2001	Observational survey Medium risk of bias Representative	*Chronic pain condition * Any chronic widespread pain (CWP), chronic regional pain (CRP) or chronic fibromyalgia (FM) *Outcomes Reported * Quality of life: Mean Scores on SF-36 subscale (Score of 100 represents the highest level of functioning): Fibromyalgia/Wides read Pain/Regional Pain: Mental Health 58/65/78;Role-emotional 45/60/78Social Functioning 58/71/84Vitality 33/44/59General health 33/49/65Bodily pain 28/40/54Role-physical 20/45/64Physical functioning 50/63/80

Fricker-Pain in Europe 2003 [33], (PowerPoint presentation—a*dditional data for Breivik 2006 available for Sweden only) *	Same cohort as Breivik 2006 *n* = 2563 for prevalence outcome *n* = 300 for all other outcomes (interviewees from Sweden) Mean age 51.5 yrs, 46% women *Target Population * Adult general population ≥18 years	Observational survey High risk of bias Representativeness unclear	*Chronic pain condition * Moderate to severe chronic noncancer pain lasting ≥6 months (1% reported cancer-related pain) *Outcomes reported * * Impact of pain on functioning—*pain prevents 64% of respondents from thinking or concentrating clearly; 55% feel tired all the time; 47% felt their pain impacted on their employment; 42% cannot function normally; 26% are in too much pain to take care of themselves and other people *Impact of pain on depression:* 24% had a diagnosis of depression by a clinician. Presence of symptoms: depression: 40.6%, insomnia: 34.8%, nervousness: 17.8% *Impact of pain on isolation and helplessness.* Feeling helpless: 55%, feeling alone: 34% *Proportion who had tried prescription medication and then stopped: *36% *Participants' reporting of duration and severity of pain: *36% had pain so severe that no more could be tolerated, 33% had chronic pain all the time, 21% had chronic pain several times a week, 18% could tolerate somewhat more pain, 2% could tolerate a lot more pain *Most common causes of chronic pain*: arthritis/OA: 27%, traumatic injury: 19%, nerve damage: 17%, cancer: 13%, herniated/deteriorating discs: 12%, tumours: 6%, fracture/deterioration of spine: 6%, carpal tunnel syndrome: 5%, RA: 5%, whiplash: 4%

Cöster et al. 2008 [34], Sweden	Sampled *n* = 9952, responders *n* = 7637, with chronic pain *n* = 290. Mean age with chronic pain: 56, 78% women *Target population * General population aged 18–74 yrs in the county of Östergötland, Sweden	Observational study Low risk of bias Representative	*Chronic pain condition * Any chronic widespread fibromyalgia pain (FM) Any general chronic widespread non-fibromyalgia pain (non-FM). Cancer pain not excluded *Outcomes reported * * Physical functioning using Arthritis Self-Efficacy subscale functioning (0–100 high score indicates better functioning); Fibromyalgia Impact subscale physical function (0–100 higher score indicates worse functioning); SF-36 subscale physical function (0–100 higher score indicates better functioning:* SF-36 subscale physical function; 52.9 (17.7) versus 65.0 (22.5) mean (SD) for FM compared with non-FM: Arthritis Self-Efficacy subscale functioning—65.7 (19.2) versus 74.8 (19.6), Fibromyalgia Impact subscale physical function 37.5 (20.3) versus 31.3 (22.6) Self-rated depressive symptoms (Beck's depression inventory*); self-rated anxiety symptoms (Beck's anxiety inventory: range 0–63, higher score more anxiety): chronic fibromyalgia pain—mean (SD) depression: 12.3 (8.0), mean (SD) anxiety: 13.2 (9.7) *Chronic non-fibromyalgia pain*—mean (SD) depression: 8.9 (5.9), mean (SD) anxiety: 9.3 (7.0) *Multidimensional pain inventory (MPI)—support (perceived support from significant others: from 0 never to 6 very often): chronic fibromyalgia pain—*mean (SD) support: 3.5 (1.7), *chronic non-fibromyalgia pain—*support: 3.5 (1.7) *Percentage of participants on sickness benefits*: with FM pain: 37.1%, with non-FM pain: 12.7%

Demmelmaier et al. 2008 [35], Sweden	*N* = 1815, mean age not reported, 41.8% men *Target population * Adults aged 20–50 yrs taken from the general Swedish population	Observational study Low risk of bias Representative	*Chronic pain condition * Any chronic nonspecific spinal pain, including mild pain. Cancer pain not excluded *Outcomes reported * * Multidimensional pain inventory—social support: punishing responses (range 0–6), solicitous responses (range 0–6), and distracting responses (range 0–6): any chronic nonspecific spinal pain (3–12 mo)—*mean (SD) for social support: punishing resp. 0.8 (1.1), solicitous resp. 3.0 (1.6), distracting resp. 3.2 (1.9) *Pain duration: any chronic non-specific spinal pain (>12 mo)—*Mean (SD) 24% of people reported being in pain for between 3 and 12 months and 76% had pain for over 12 months pain intensity. On a 1–100 score (100 being most pain), the mean pain score was 44.8 (SD18.6) for the <12 month group, and 47.3 (SD 17.7) for the >12 month group *Mean pain intensity (SD) on a 1–100 scale: *punishing resp. 0.8 (1.1), solicitous resp. 2.5 (1.5), distracting resp. 2.7 (1.8) *Pain duration and intensity 3–12 months (depression comorbidity). *Pain 3–12 months: mean score 44.8 (SD 18.6), pain >12 months: 47.3 (SD 17.7) *HADS-D score (hospital anxiety and depression rating scale of 0–14): *mean score 47.3 (SD 17.7) Pain 3–12 months: mean score 44.8 (SD 18.6), pain >12 months Mean score 47.3 (SD 17.7) *pain duration >12 months (depression comorbidity). *depression: HADS-D mean 5.2 (SE 0.27); Depression: HADS-D mean 5.3 (SE 0.15), those with chronic nonspecific spinal pain had a significantly higher HADS-D mean score then those with recurrent nonspecific pain lasting <3 months (*n* = 215; 4.2 [SE 0.24]; *P* < 0.05). *Mean BMI score pain duration 3–12 months: *BMI mean 25.1 (SD 4.0) *Mean BMI score pain duration >12 months: *BMI mean 24.5 (SD 3.6)

Ekman et al. 2005 Spine [36], Sweden	*N* = 302, mean age 48 yrs, 47% men *Target population * Adult general population of Sweden ≥18 years	Observational survey and economic study High risk of bias Not representative	*Chronic pain condition * Any chronic noncancer low back pain (LBP) *Outcomes reported * * Costs to society: direct costs per patient *Total average annual direct cost per patient: €3089 (15% of total costs), total healthcare costs when home help was excluded: €3017 (14.7% of total costs) *Indirect costs. *Largest indirect cost: sickness absence from work—average yearly cost per patient: €9563 *Largest indirect cost item and total indirect costs:* total indirect costs per patient: €17,576 (85% of total LBP costs); total annual costs per patient (estimated): €20,666 *Satisfaction with treatment, that is, pain relief, tolerance, and overall treatment: median of responses on a scale from 1 (very dissatisfied) to 6 (very satisfied), *Patients scored a median of 3 (somewhat dissatisfied) for all 3 questions—pain relief, tolerance, and overall treatment. *Drug treatments reported:* step 1: NSAIDs 51%; COX-2 inhibitors 5%, analgesics: 59%, muscle relaxants/anxiolytics: 11%, antidepressants: 8%, other: 1%

Gerdle et al. 2004 [37], Sweden	*N* = 7637, median age 46 yrs, 47% men Adults aged 18–74 yrs in the county of Östergötland in southern Sweden	Observational survey High risk of bias Representativeness unclear	*Chronic pain condition * General chronic pain *Outcomes reported * * Proportion of patients seeking health care for their pain: *64.8%, of those in constant pain: 86.3%, of those with severe pain: 79.5%

Guez et al. 2003 [38], Sweden	Responders *n* = 4392, with chronic pain *n* = 814, mean age 51 yrs, 39% men *Target population * Adults aged 25–64 yrs resident in Northern Sweden	Observational survey Medium risk of bias Representative	*Chronic pain condition * Any chronic neck pain (cancer not excluded) *Outcomes reported * * % on sick leave due to neck pain: *29% *Prevalence of chronic neck pain: *18.5%

Gummesson et al. 2003 [39], Sweden	Responders *n* = 2466, mean age 50 yrs, 46% men Adults aged 25–74 yrs from Southern Sweden	Observational survey Medium risk of bias Representative	*Chronic pain condition * Any chronic upper extremity pain; any chronic upper extremity pain associated with physical impairment (cancer not excluded) *Outcomes reported * *Prevalence of chronic upper extremity pain with physical impairment: *20.8% (95% CI 19.2, 22.5) *Cooccurrence with chronic upper extremity numbness or tingling:* 32% (164/513) of those with chronic pain with physical impairment or 6.7% (95% CI 5.7, 7.7) of total sample (164/2466)

Jacobsson et al. 2007 [40], Sweden	Analysed responders *n* = 613, mean age 66 (SD 14.1), 74% women *Target population * Adults in Malmö area of Sweden with rheumatoid arthritis.	Observational survey Medium risk of bias Representative	*Chronic pain condition * Chronic noncancer pain (rheumatoid arthritis pain) *Outcomes reported * * Percentage of patients with RA on sick leave or sickness pension *Women: 22%, men: 16%, short-term sick-leave: 6.5%, long-term sick-leave: 8.5%, On early retirement due to RA: 18.4%, total retired: 47%; total on sick pension or sick leave: 21% *Mean annual total costs per patient: *mean annual total costs per patient: 108,370 SEK (€12,286) *Annual direct costs.* Annual direct costs: 44,485 SEK (€5,043) (41% of the total costs), direct healthcare costs. 33,092 SEK (€3,751) (30.5% of the total costs) *Costs to patients. *Costs to patients: 4302 SEK (€488) (4% of total costs)—this included costs of informal care (2.5% of total) and costs of private investments (1.5% of total costs) *Duration of RA.* All: mean 16.7 yrs (SD12.9), women: mean 16.8 yrs (SD 12.9); men: mean 16.3 yrs (SD 12.7) *Patients' perception of pain on a 100-point VAS scale (over the last week). *All: mean score 40 (SD 24), women: mean score 42 (SD 24); men: mean score 35 (SD24)

Jakobsson et al. 2004 [41], Sweden	Age stratified sample *n* = 8500, selected population *n* = 294, age range 76–100 yrs, 34.4% men *Target population * Elderly population ≥75yrs in Southern Sweden	Observational survey High risk of bias Not representative	*Chronic pain condition * Any chronic general pain, including mild pain in the elderly (≥75 yrs). Cancer pain was not excluded *Outcomes reported * * Level of social support in the elderly with chronic pain Multidimensional pain inventory—subscale social support (range 0–6, 6 indicates high degree of support): *support (mean (SD)—living at home: 3.83 (2.10), living in special accommodation: 3.64 (1.92), living alone: 3.29 (2.05), living with someone: 4.69 (1.79) *Percentage that did not use any pain-relieving method:* 3.8% *Percentage using prescription and nonprescription medication in various environments and helpfulness of treatment: helpfulness calculated by median score [75th–25th percentile]*: living at home—39% used prescription medication, helpfulness: median 3.0 [4.0–2.0] generally helpful, 23% used nonprescription medication Helpfulness: median 3.0 [4.0–2.0] generally helpful living in special accommodation—31% used prescription medication Helpfulness: median 2.5 [3.0–2.0] somewhat-generally helpful, 9% used nonprescription medication, helpfulness: median 2.0 [3.3–1.0] somewhat helpful Living alone—51% used prescription medication, helpfulness: median 3.0 [4.0–2.0] generally helpful, 28% used nonprescription medication Helpfulness: median 2.5 [4.0–2.0] somewhat-generally helpful Living with someone—44% used prescription medication; helpfulness: median 3.0 [4.0–3.0] generally helpful' 21% used nonprescription medication, helpfulness: median 2.5 [3.3-2.0] somewhat-generally helpful *Cause of pain in elderly with any chronic general pain: *37% did not know cause of pain *Of those that received a diagnosis or knew cause of pain: *osteoarthritis 34%, musculoskeletal diseases/problems 27%, nonmusculoskeletal diseases/problems 16%, other rheumatic diseases 14%, rheumatoid arthritis 6%, osteoporosis 2%, unspecified musculoskeletal pain 1%

Kato et al. 2006 [42], Sweden	Sampled *n* = 44,897, mean age 59.8 yrs (SD 11.1), 46.5% men *Target population * All twins born in Sweden between 1886 and 1959 (registry based)	Observational case-control study Low risk of bias Representative	*Chronic pain condition * Any chronic widespread pain (CWP) *Outcomes reported * * Prevalence of chronic widespread pain: *4.1% *CWP in those aged => 42 yrs: *poor general health was found in 83.1% of participants with CWP and 26.7% of participants without CWP *Quality of life:* health which prevents activities was reported in 81.9% of people with CWP and 26.4% of participants without CWP *Depression:* current depressive symptoms: 40.2%, lifetime major depression: 36.2%, lifetime generalized anxiety disorder: 9.9%, lifetime eating disorders, aged <65 yrs: 38.3% * Comorbidity with CWP. Cotwin MZ analysis: (OR (95% CIs) adjusted for age and sex):* chronic impairing fatigue, age ≤64 yrs: 3.71 (2.06–6.70), CFS-like illness, age ≤64 yrs: 9.75 (3.48–27.28), Joint pain (i.e., ≥1 of RA, prolonged joint pain, or OA): 4.60 (2.63–8.04), possible RA: 3.89 (1.87–8.09), prolonged joint pain: 5.56 (2.73–11.30), OA, knee or hip: 2.43 (1.30–4.53), migraine, age ≤64 yrs: 3.27 (1.67–6.43), tension-type headache, age ≤64 yrs: 3.00 (1.47–6.14), Current depressive symptoms: 2.00 (1.27–3.15), lifetime major depression: 1.13 (0.74–1.72), lifetime generalized anxiety disorder: 1.60 (0.73–3.53) lifetime eating disorders, age ≤64 yrs: 0.93 (0.59–1.45), irritable bowel syndrome: 3.50 (1.84–6.65), GERD: 2.17 (1.33–3.56), urinary tract problems: 1.77 (1.05–2.99), prolonged cough, >3 mo: 1.41 (0.76–2.63) Possible asthma: 1.25 (0.65–2.41), allergy, any: 1.70 (1.08–2.67) obesity, BMI ≥30: 2.09 (1.02–4.29), overweight, BMI ≥25: 1.28 (0.76–2.16) sleep problems, age ≥55 yrs: 3.00 (1.09–8.25), poor general health: 6.20 (3.59–10.70), health prevents activities: 5.22 (3.15–8.65), poorer health status than 5 yrs ago: 3.27 (2.11–5.07), frequent infections, >2 per year: 3.08 (1.61–5.91)

Mullersdorf and Soderback 2000 Int J Rehab [43], Sweden	Invited *n* = 10,000, responders *n* = 6490. Mean age not reported, 48% men *Target population * Swedish population aged 18–58 yrs	Observational survey High risk of bias Representativeness unclear	*Chronic pain condition * Any general long-term pain (defined as at least 3 months). Cancer was not excluded *Outcomes reported * * % with self-perceived activity limitation and/or participation restriction due to pain: *In participants with long-term pain: 16.6%, men 7.4%; women 9.3%; In participants with long-term and recurrent pain. 13.0%; men 5.6%; women 7.3%

Müllersdorf and Söderback 2000 Dis & Rehab [44], Sweden	Invited *n* = 10,000, study sample (pain sufferers) *n* = 1305, control group *n* = 150. Mean age not reported, 48% men *Target population * Swedish population aged 18–58 yrs	Observational survey Low quality High risk of bias Representativeness unclear	*Chronic pain condition * Any general chronic pain—long-term pain and recurrent pain *Outcomes reported * * % of people on/not on sick leave over last year. Not* on sick leave over the past year: 44.3%, on sick leave for <3 months: 40.6%, on sick leave for >3 months: 15.1%, mean sick leave: 43 days *Frequencies (N) of health care staff that respondents consulted and received treatment from and % differences between genders: *physicians (966) M 74% F 74%, physiotherapist (722) M 49.3%, F 59.0%, chiropractor (397) M 30.8%, F 30.2%, nurse (257) M 22.7%, F 17.8%, occupational therapist (139) M 6.0%, F 13.5%, psychologist (97) M 5.2%, F 8.8%, welfare officer (79) M 3.0%, F 7.9%, vocational guidance officer (46) M 3.4%, F 3.6%, clergymen, lay worker, priest (22) M 2.0%, F 1.5%, other (60) M 3.4%, F 5.3%

Norrbrink Budh and Lundeberg 2004 [45], Sweden	*N* = 130, at followup 3 years later, *n* = 123, responders *n* = 101. Mean age 55.3 yrs, 49% men *Target population * Adults with spinal cord injuries at a hospital in Stockholm, Sweden	Longitudinal study Medium risk of bias Not representative	*Chronic pain condition * Any chronic pain due to spinal cord injury (SCI) *Outcomes reported * * Percentage using drug and using or tried nondrug treatments: *70.5% *Percentage not using drugs at time of study:* 51% *Percentage of above pts who tried one or more analgesics:* 41.2% *Participants with the following characteristics more likely to try nondrug therapy for pain relief:* those with moderate pain (VAS 40–69 mm) versus mild pain (VAS 0–39 mm): adjusted OR 4.94 (95% CI 1.5, 16.7). Those with severe pain (VAS ≥70 mm) versus mild pain (VAS 0–39 mm): adjusted OR 10.45 (95% CI 2.0, 54.7). Those with aching pain (adjusted OR 4.04 [95% CI 1.3, 12.8]) and cutting/stabbing pain (adjusted OR 3.55 [95% CI 1.1, 11.1]) were also more likely to try nondrug treatment. *Percentage using drug treatments: *48.9% *Percentage types of drugs used. *Step I—NSAIDs: 15.6%, step III (opiates): 34.4% anti-convulsants: 12.2% anti-depressants: 11.1% *% tried or using nondrug treatments:* acupuncture: 35.6%, massage: 34.4%, TENS: 32.2%, heat: 24.4%, cold: 10.0%, other (mental training): 5.6%, other (physical training): 4.4%

Raak et al. 2003 [46], Sweden	*N* = 53, with fibromyalgia *n* = 32; mean age 46 yrs, 100% female *Target population * Adult women aged 30–68 yrs in the area of Linköping, Sweden	Observational study Low quality High risk of bias Representativeness unclear	*Chronic pain condition * Any chronic fibromyalgia (FM) *Outcomes reported * * Costs to society: *working part time/full time/retired: 21%, partly sick listed: 24%, *Employment status of patients with FM:* sick listed/sickness pension: 55%

Silvemark et al. 2008 [47], Sweden	*N* = 294, mean age 38.1 yrs (SD9.4), 34% men *Target population * Adult population of Upsala, Sweden, aged 18–64 yrs	Observational survey Medium risk of bias Representativeness unclear	*Chronic pain condition * Any long-term noncancer pain *Outcomes reported * * Satisfaction with social contacts: *32% satisfied, 68% dissatisfied *Proportion of participants on sickness benefits.* on sickness benefit: 69%, on sickness pension: 4%, on social allowance: 1%

Simonsson et al. 1999 [48], Swedene	Sampled *n* = 3928, responders *n* = 2425. Mean age and gender not reported. *Target population *Adult general population of Sweden aged 20 to 74 yrs.	Observational study Low risk of bias Representative	*Chronic pain condition * Any rheumatoid arthritis (including inactive disease) *Outcomes reported * * Prevalence of rheumatoid arthritis (RA) in Sweden:* 0.51% (95% CI 0.31–0.79)

*Study [33] gives additional patient information on the cohort in Study [2] so these were classed as one study.
