# Supplementary material for: Epidemiology of Chronic Pain in Denmark and Sweden
Source: Pain Res Treat. 2012 May 23;2012:371248. doi: 10.1155/2012/371248 (PMC3366230; doi:10.1155/2012/371248)
Supplement: Supplementary file 1 — Appendix 1 is the full Medline Strategy we utilised for the searches. This strategy was adapted specifically for each subsequent database and the keywords associated with epidemiology and chronic pain were adapted according to the thesaurus and configuration of each individual database. Appendix 2 is a list of clinical questions that were addressed systematically in our literature review. The reviews were initially conducted separately with each question being applied for each country. Where results are not presented for a particular question, this is because either (a) no papers were found addressing the question or (b) information/data found was not considered to be of appropriate study quality or relevance to the review. Appendix 3 describes the detailed quality assessment criteria which we used to assess each included study. All studies were assessed on all eight criteria/questions, and finally a score was generated (see section 9) to assign a risk of bias for each study i.e. low, medium or high risk. Appendix 4 contains the results of the quality assessment for each included study from Denmark and Sweden. The parameters 1-8 reflect the answers/scoring to Questions 1-8 on the quality assessment measure (see Appendix 3). The final column shows the risk of bias assessed for each individual study. [file 371248.f1.pdf]

Epidemiology of Chronic Pain in Denmark and Sweden – Supplementary Materials

Appendix 1 –Electronic Strategy (reported here for Medline only)

Search to identify epidemiological literature on chronic pain in Europe

Searches were carried out to identify epidemiology literature published on this topic. The strategy was limited to specific European countries and corresponding languages (German, French, Swedish, Spanish, Italian, Dutch, English, and Danish).
Databases were searched from 1995-2009.

The following databases were searched:

- MEDLINE (1995-2009/08/wk 3) (OvidSP)
- MEDLINE In-Process Citations (1995-2009/08/31) (OvidSP)
- Embase (1995-2009/wk 35) (OvidSP)
- Cochrane Database of Systematic Reviews (1995-2009, Cochrane Library, Issue 3:2009) (internet)
- CENTRAL (1995-2009, Cochrane Library, Issue 3:2009) (internet)
- Database of Abstracts of Reviews of Effects (DARE) (1995-2009/09/02) (internet)
- Health Technology Assessment Database (HTA) (1995-2009/09/02) (internet)
- Guidelines International Network database (1995-2009/09/02) (internet)

A total of 20317 references were retrieved.
Following deduplication, 16619 references were screened for inclusion.

MEDLINE (2006/07-2008/01/wk1) (OvidSP)

The Medline search was from 1995 to 2009/08/wk3 and identified 7544 references.

1 ((Chronic$ or longterm or long term or sustained or long standing or permanent$ or intractable$ or persistent$ or unremitting or unrelenting or continual$ or continuous$ or constant$ or unending or unceasing) adj3 (back$ or muscl$ or neck or shoulder$) adj3 (pain or pains or painful$ or sore$ or tender$ or discomfort or

ache$ or aching or strains or strained or sprain or sprains or sprained or injur$ or damag$)).ti,ab. (5549)

2 Diabetic Neuropathies/ or exp polyneuropathies/ or exp Mononeuropathies/ (41009)

3 (neuropath$ or arthralg$ or neuralg$ or fibromyalg$ or DPN or mononeuropath$ or polyneuropath$ or nerve$ pain$).ti,ab. (86784)

4 exp osteoarthritis/ or Arthritis, Rheumatoid/ or (rheumatoid arthrit$ or osteoarthrit$).mp. (114752)

5 or/2-4 (225697)

6 exp Pain/ or (pain or pains or painful$).ti,ab. (412550)

7 5 and 6 (38390)

8 exp muscle, skeletal/ or muscl$.ti,ab. (485234)

9 exp Pain/ or (pain or pains or painful$ or sore$ or tender$ or discomfort or ache$ or aching or strains or strained or sprain or sprains or sprained or injur$ or damag$).ti,ab. (1275222)

10 8 and 9 (60857)

11 chronic disease/ (187455)

12 ((longterm or chronic$ or long term or sustained or long standing or permanent$ or intractable$ or persistent$ or unremitting or unrelenting or continual$ or continuous$ or constant$ or unending or unceasing) adj2 (disorder$ or condition$ or illness$ or illhealth$ or ill health$ or malad$ or sickness or disease$)).mp. (279413)

13 or/11-12 (279413)

14 13 and 6 (26387)

15 ((Chronic$ or longterm or long term or sustained or long standing or permanent$ or intractable$ or persistent$ or unremitting or unrelenting or continual$ or continuous$ or constant$ or unending or unceasing) adj2 (pain or pains or painful$)).ti,ab. (26482)

16 pain, intractable/ or pain, referred/ (4967)

17 exp Back Pain/ (24299)

18 exp neuralgia/ (9698)

19 Neck Pain/ (2922)

20 exp Arthralgia/ (4534)

21 Fibromyalgia/ (4394)

22 low$ back pain$.mp. (17092)

23 or/15-22 (71324)

24 or/1,7,10,14,23 (160107)

25 Demography/td, sn [Trends, Statistics & Numerical Data] (5)

26 exp Patient Compliance/ (38051)

27 Attitude to Health/ (60831)

28 "Delivery of Health Care"/ (50846)

29 health surveys/ or health care surveys/ or questionnaires/ or morbidity/ or prevalence/ (370362)

30 "Quality of Health Care"/ (43847)

31 Professional Practice/ (13119)

32 Public Health Practice/ (2930)

33 epidemiologic studies/ or cohort studies/ (105554)

34 Epidemiology/ (10744)

35 Health Services/ (16293)

36 Drug Utilization/ (13754)

37 exp data collection/ (1073659)

38 Interview/ (20219)

39 Interviews as Topic/ (26089)

40 Disability Evaluation/ (25463)

41 "Costs and Cost Analysis"/ (37661)

42 Insurance/ (2709)

43 Patient Satisfaction/ (41604)

44 case-control studies/ or cross-sectional studies/ (213595)

45 (morbidit$ or frequency or frequencies or occurrence$ or incidence$ or prevalence$ or number$ or times or rate or rates or episode$ or natural history or epidemiolog$ or survey$).ti,ab. (3560617)

46 ((therap$ or treatment$ or intervention$ or medicat$ or drug or drugs or medicine$ or regime$) adj3 (discontinu$ or ceas$ or drop$ out or adher$ or continu$ or pattern$ or complian$ or comply$ or complies or terminat$ or halt$ or durat$ or

persist$ or stop$ or withdraw$ or suspend$ or suspension$ or break$ off or attrition)).ti,ab. (129527)

47 (awareness or impact).ti,ab. (318546)

48 or/25-47 (4587719)

49 24 and 48 (62755)

50 (german or french or swedish or spanish or italian or dutch or danish).lg. (2067881)

51 49 and 50 (4641)

52 exp Europe/ or (france or french or german or germany or italy or italian or spain or spanish or catalan or sweden or swedish or england or english or britain or british or united kingdom or uk or scotland or scottish or wales or welsh or ireland or irish or netherlands or holland or dutch or danish or denmark).ti,ab. (1094356)

53 24 and 48 and 52 (6126)

54 english.lg. and 53 (5042)

55 51 or 54 (9668)

56 limit 55 to yr="1995 -Current" (7544)

<u>Appendix 2 - Questions to be addressed</u>

<u>Epidemiology flow</u>

1. What are the population and demographics of Denmark?
2. What is the prevalence of chronic pain conditions?
3. What is the incidence of chronic pain conditions?
4. What percentage of chronic pain patients are untreated or inadequately treated?
5. How many chronic pain patients present themselves for treatment?
6. How many chronic pain patients get treated broken down by treatment?
7. What is the compliance of treated chronic pain patients?

<u>Questions leading to in depth information to the numbers mentioned in the Epidemiology flow</u>

8. What is the disease duration of chronic pain conditions?
9. What are the demographics of pain sufferers?
10. What are the co-morbidities of pain sufferers?
11. How many sufferers have inadequate pain control?
12. What is the impact of chronic pain on:
    a. Quality of life
    b. Activities of daily living
    c. Depression and other mental illness
    d. Isolation, helplessness
    e. Days off work
    f. Incapacity benefits
13. What are the costs of chronic pain from a
    a. Societal perspective?
    b. Health care system perspective?
    c. Patient perspective?
14. What are issues/determinants of patients' awareness of chronic pain?
15. What are issues/determinants of health care professionals' awareness of chronic pain?
16. What are the main symptoms and complaints with which patients present themselves to health care professionals?
17. What are the frequencies of drug (per WHO class), non-drug, and combined treatments?
18. What are determinants of treatment choice between drug treatment and non-drug treatment?
19. What are determinants of treatment choice within drug treatments?
20. What are determinants of compliance / adherence to drug treatments?
21. What is patients' satisfaction about drug treatments?

Appendix 3 . Guide for assessing the quality of observational studies (devised by the authors using STROBE guidelines as a reference) [16, 57, 59].

1. **Was there an adequate description of study design and setting? Y, N or unclear**

   As a minimum, authors should have described the design (e.g. retroactive study of patient records, telephone or postal cross-sectional survey, etc) **and** described the setting (e.g. pain clinics, population registered at general practices, medical records database, etc) **along with** relevant dates (periods for recruitment, follow-up, data collection, etc).

   If they did not report all of the above - the description was inadequate.

   Select Unclear if authors reported design and setting information but it was presented unclearly or incompletely (e.g. the number of general practices was not reported or only the recruitment start date was reported, etc)

2. **Was there an adequate description of eligibility criteria? Y, N or unclear**

   As a minimum, authors should have described inclusion/exclusion criteria (e.g. chronic pain patients had to have pain continuously for at least 3 months, cancer pain was excluded, etc) **and** they should have taken some step to confirm the diagnosis or, in the case of interviewed health professionals, the authors should have provided confirmation that the doctors' patients were correctly diagnosed (e.g. the authors conducted a physical examination or cross-checked with a patient register to confirm diagnosis).

   If they did not give **any** inclusion/exclusion criteria **and** they did not confirm the diagnosis (e.g. authors did not report that arthritic pain patients were

examined to confirm pain was due to arthritis) then the description was inadequate.

Select Unclear if authors reported eligibility criteria but it was presented unclearly or they did not confirm the diagnosis (e.g. chronic pain was an inclusion criterion but chronic pain was not defined or they did not confirm the included participants had chronic pain).

3. **Is the study population representative of the target population? Y, N, Unclear**

Note – for this question, the target population is the population studied in the study, not the population that we are studying for this project (e.g. if study examines the prevalence of chronic back pain in nurses, this question asks whether the study population was likely representative of a nursing population)

The authors should have described how the sample size was arrived at **and** how the patients were selected (e.g. consecutive vs. non-consecutive patients entering pain clinic, random postal or telephone survey) **and** the demographics of the sample should have been described as comparable to the target population. For surveys, an attempt should have been made to compare non-responders to responders.

If the authors reported the above **and** there is good reason to doubt that the population was representative (e.g. significantly more women than men responded to a postal survey when compared to the non-responder group and the results were not adjusted for this) then the population was not representative.

Select Unclear if the authors did not report or discuss comparability with their target population (e.g. a telephone survey of the general population but

authors did not state whether demographics of sample were comparable to the general population)

4. **Is there an adequate description of outcomes? Y, N, Unclear**

Note – as there are as many outcomes as there are questions, this question must necessarily reflect all the outcomes measured in the study

If authors generally described how they measure the outcome **and** clear definitions were given for key terms (e.g. one year prevalence, incidence per 100 000, what they meant by adequate treatment, untreated, etc.) then the outcomes were adequately described.

If authors failed to describe how they measured their outcomes **and** if they failed to describe or qualify key terms (e.g. they measured prevalence but did not qualify it as life time, year long, etc) then the outcomes were not adequately described.

Select Unclear if the descriptions or definitions were unclear (e.g. authors described patients as inadequately treated but did not provide the standard of treatment for comparison – like a clinical guideline)

5. **Is there an adequate description of statistical methods? Y, N, Unclear**

If the authors described their statistical methods **and** described potential confounders or effect modifiers **and** how they were dealt with, then the statistical methods were adequately described

If the authors failed to describe all of the above then the description of statistical methods was inadequate

Select Unclear if the authors described their methods but it was difficult to ascertain exactly how or why they used their methods.

6. **Is there an adequate description of the study participants? Y, N, Unclear**

If the authors provided **more** than just age and gender (e.g. pain duration, occupations, pain type, etc.) then the participants were adequately described

If authors **only** provided age and gender then the description was inadequate

Select Unclear if the population descriptions were unclear (e.g. numbers in texts and figures didn't match or add up).

7. **Was there an adequate description of losses to follow-up (plus – were losses to follow-up too high)? Y, N, Unclear, Not Applicable**

If authors clearly described the losses to follow-up or if the loss was <10% by 12 months and <25% for periods longer than 12 months, then this was adequate

If authors did not describe the losses to follow-up or if the loss is >10% by 12 months and >25% for periods longer than 12 months, then this was inadequate

Select Unclear if authors described losses to follow-up but it was difficult to follow, incomplete or the numbers in the text do not match figures.

Select Not Applicable if there was no follow-up (i.e. not a longitudinal study).

8. **Are the results reported as unadjusted or confounder adjusted? Y, N, Unclear**

This question is asking whether the authors reported what was done – i.e. is it clear whether the reported results were adjusted or not?

Answer Yes, if authors clearly reported their results as unadjusted or confounder adjusted (or equivalent language – univariate, multivariate) **and** they provided precision (e.g. SE or SD, CIs). Authors should also have indicated what confounders were adjusted for and why they were included.

Select No if authors **did not indicate** whether results were unadjusted or confounder adjusted – i.e. they did not report what was done.

Select Unclear if authors reported results as unadjusted or confounder adjusted **but** no precision was given **or** the results were otherwise unclearly presented

9. **Overall score:**

High (**Low risk of bias)** – all criteria met (8 Yes's or 7 Yes's if not a longitudinal study)
Medium (**Medium risk of bias)** – 2 to 3 criteria not met (i.e. 2-3 No's or Unclear)
Low **(High risk of bias)** – 4 or more criteria not met (i.e. ≥4 No's or Unclear)

Appendix 4 – Quality assessment of selected studies for Denmark and Sweden

Study ID	Country	1	2	3	4	5	6	7	8	Overall Risk of bias/Quality
Breivik 2006/Fricker 2003 (same cohort) **[2/3]**	Denmark and Sweden	Y	N	UC	Y	N	Y	NA	N	High bias risk (Low quality)
Ekholm 2009 **[17]**	Denmark	Y	Y	Y	Y	Y	Y	NA	Y	Low bias risk (High quality)
Eriksen 2003 **[18]**	Denmark	Y	Y	UC	Y	Y	Y	NA	Y	Low bias risk (High Quality)
Eriksen 2004EJP **[25]**	Denmark	Y	Y	UC	Y	N	Y	NA	UC	Medium bias risk (Medium quality)
Eriksen 2004P **[21]**	Denmark	Y	N	UC	Y	Y	Y	Y	Y	Medium bias risk (Medium quality)
Eriksen 2006 **[22]**	Denmark	Y	N	UC	Y	Y	Y	NA	Y	Medium bias risk (Medium quality)
Højsted 1999 **[23]**	Denmark	Y	N	UC	Y	Y	Y	NA	N	Medium bias risk (Medium quality)
Jensen 2004 **[19]**	Denmark	Y	Y	Y	Y	Y	Y	NA	Y	Low bias risk (High quality)
Kronborg 2009 **[24]**	Denmark	Y	Y	UC	Y	Y	Y	NA	N	Medium bias risk (Medium quality)
Sjøgren 2009 **[20]**	Denmark	Y	Y	Y	Y	Y	Y	NA	Y	Low bias risk (High quality)
Thomsen 2002 **[26]**	Denmark	Y	Y	UC	Y	UC	UC	Y	N	High bias risk (Low quality)
Andersson 1999 J Epi Comm **[27]**	Sweden	Y	N	Y	Y	Y	N	NA	Y	Medium bias risk (medium quality)

Study ID	Country	1	2	3	4	5	6	7	8	Overall Risk of bias/Quality
Andersson 1999 Scand J PHC **[28]**	Sweden	Y	N	Y	Y	Y	N	NA	Y	Medium bias risk (medium quality)
Arvidsson 2008 **[29]**	Sweden	Y	N	Y	Y	Y	Y	Y	Y	Low bias risk (High quality)
Ben-Menachem 1995 **[30]**	Sweden	N	U	U	Y	N	Y	NA	N	High bias risk (Low quality)
Bergman 2001 **[31]**	Sweden	Y	N	Y	Y	Y	Y	NA	Y	Low bias risk (High quality)
Bergman 2005 **[32]**	Sweden	N	Y	Y	Y	U	Y	NA	Y	Medium bias risk (Medium quality)
Fricker 2003 (PIE) (*Powerpoint – Sweden only*) **[33]**	Sweden	Y	N	U	Y	N	Y	NA	N	High bias risk (Low quality)
Cöster 2008 **[34]**	Sweden	Y	Y	Y	Y	Y	Y	NA	Y	Low bias risk (High quality)
Demmelmaier 2008 **[35]**	Sweden	Y	N	Y	Y	Y	Y	NA	Y	Low bias risk (High quality)
Ekman 2005 **[36]**	Sweden	N	N	U	Y	U	Y	NA	U	High bias risk (Low quality)
Gerdle 2004 **[37]**	Sweden	Y	N	N	Y	U	Y	NA	U	High bias risk (Low quality)
Guez 2003 **[38]**	Sweden	Y	N	Y	Y	U	Y	NA	Y	Medium bias risk (Medium quality)
Gummesson 2003 **[39]**	Sweden	Y	N	Y	Y	Y	N	NA	Y	Medium bias risk (Medium quality)
Jacobsson 2007 **[40]**	Sweden	Y	Y	Y	Y	N	Y	NA	N	Medium bias risk (Medium quality)
Jakobsson 2004 **[41]**	Sweden	U	N	N	Y	N	Y	NA	N	High bias risk (Low quality)
Kato 2006 **[42]**	Sweden	Y	N	Y	Y	Y	Y	NA	Y	Low bias risk (High quality)

Study ID	Country	1	2	3	4	5	6	7	8	Overall Risk of bias/Quality
Müllersdorf 2000 Int J Rehab **[43]**	Sweden	Y	N	Y	N	N	N	NA	N	High bias risk (Low quality)
Müllersdorf 2000 Dis & Rehab **[44]**	Sweden	N	U	U	Y	U	Y	NA	N	High bias risk (Low quality)
Norrbrink Budh 2004 **[45]**	Sweden	Y	N	N	Y	Y	Y	Y	Y	Medium bias risk (Medium quality)
Raak 2003 **[46]**	Sweden	N	Y	U	Y	N	Y	NA	N	High bias risk (Low quality)
Silvemark 2008 **[47]**	Sweden	Y	Y	U	Y	N	Y	NA	N	Medium bias risk (Medium quality)
Simonsson 1999 **[48]**	Sweden	Y	Y	Y	Y	Y	Y	NA	Y	Low bias risk (High quality)

1– Adequate description of study design and setting, 2 – Adequate description of eligibility criteria, 3 – Study population is representative of target population, 4 – Adequate description of outcomes, 5 – Adequate description of statistical methods, 6 – Adequate description of study participants, 7 – Adequate description of losses to follow-up, 8 – Results reported as unadjusted or confounder-adjusted N – no, NA – not applicable, UC – unclear, Y – yes
