# Supplementary material for: Epidemiology of Chronic Pain in Denmark and Sweden
Source: Pain Res Treat. 2012 May 23;2012:371248. doi: 10.1155/2012/371248 (PMC3366230; doi:10.1155/2012/371248)
Supplement: Supplementary file 2 [file 371248.f2.pdf]

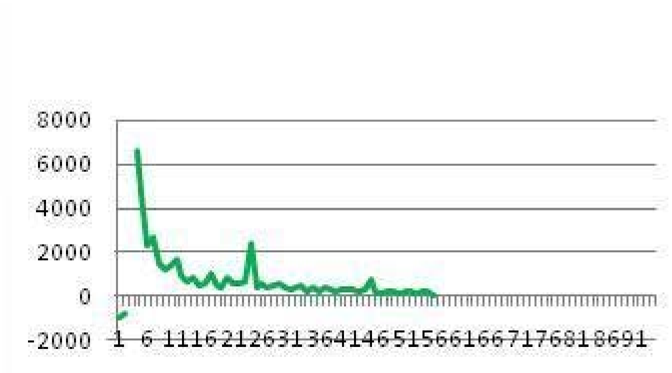

Figure 5: The time series of rehabilitation group BI baseline unit cost effectiveness.

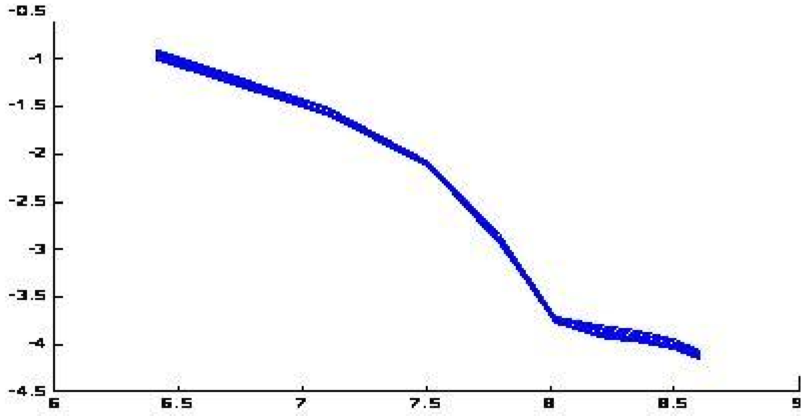

Figure 6: The fitting chart of Chinese medicine group for Fugle-Meyer baseline fractal m _ 2.

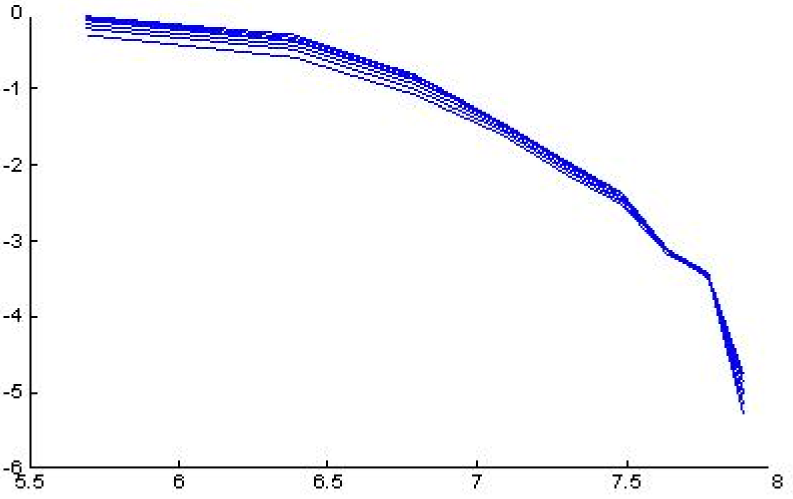

Figure 7: The fitting chart of Chinese medicine group for NIHSS baseline fractal m _ 2.

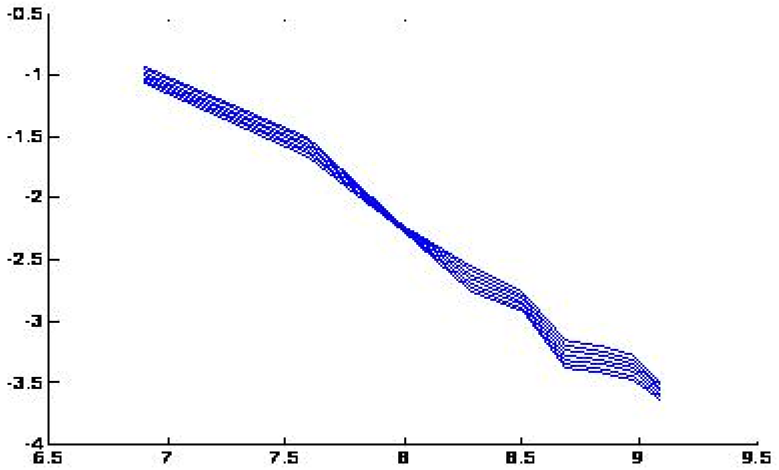

Figure 8: The fitting chart of Chinese medicine group for BI baseline fractal m _ 4.

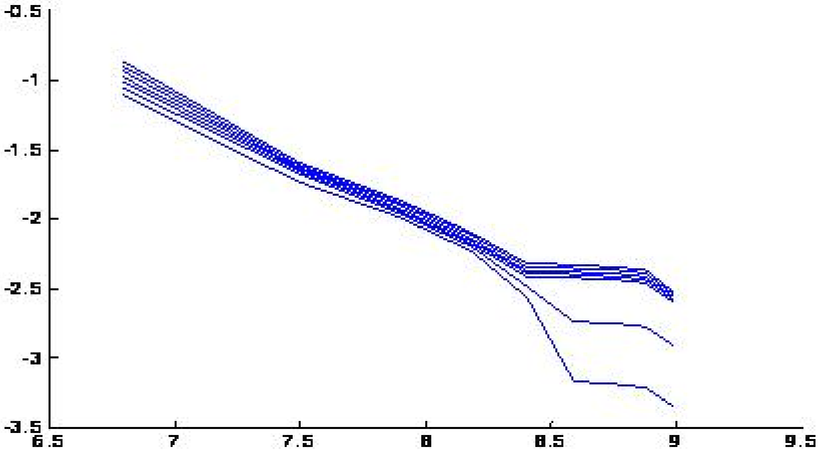

Figure 9: The fitting chart of rehabilitation group for Fugle-Meyer baseline fractal m _ 2.

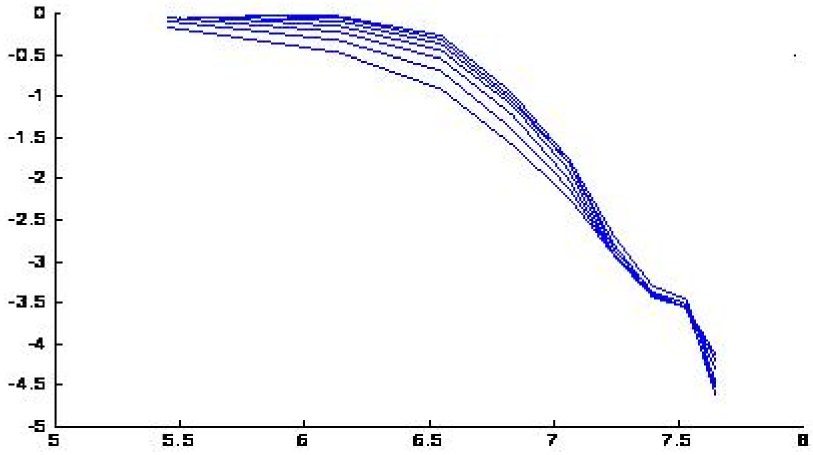

Figure 10: The fitting chart of rehabilitation group for NIHSS baseline fractal m _ 2.

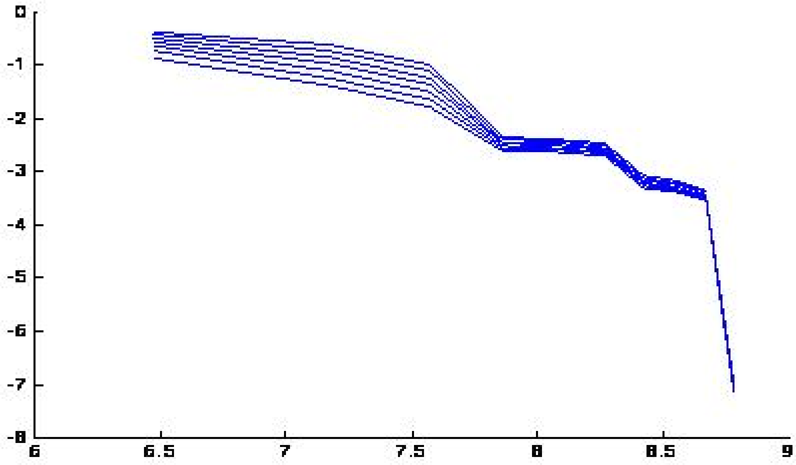

Figure 11: The fitting chart of rehabilitation group for BI baseline fractal m =4.

Table 1: Efficacy and cost effectiveness of the fractal dimension of unit table.

	Chinese medicine group	The rehabilitation group
Fugle-Meyer Baseline	−1.0720549	−0.9573777263
NIHSS Baseline	−1.000721214	−0.8529432974
BI Baseline	−1.022924504	−0.942960312
Average value	−1.0319	−0.91776

Table 2: Lyapunov time series index table of Chinese medicine group for unit cost effectiveness.

Chinese medicine group	Fugle-Meyer baseline	NIHSS baseline	BI baseline
Embedding dimension	2	2	4
Lyapunov index	0.0615	0.1362	0.0442

Table 3: Lyapunov time series index table of the rehabilitation group for unit cost effectiveness.

The rehabilitation group	Fugle-Meyer baseline	NIHSS	baseline
Embedding dimension	2	2	4
Lyapunov index	0.1372	0.6615	0.3019
